# Somatostatin Receptor PET/MR Imaging of Inflammation in Patients With Large Vessel Vasculitis and Atherosclerosis

**DOI:** 10.1016/j.jacc.2022.10.034

**Published:** 2023-01-31

**Authors:** Andrej Ćorović, Christopher Wall, Meritxell Nus, Deepa Gopalan, Yuan Huang, Maria Imaz, Michal Zulcinski, Marta Peverelli, Anna Uryga, Jordi Lambert, Dario Bressan, Robert T. Maughan, Charis Pericleous, Suraiya Dubash, Natasha Jordan, David R. Jayne, Stephen P. Hoole, Patrick A. Calvert, Andrew F. Dean, Doris Rassl, Tara Barwick, Mark Iles, Mattia Frontini, Greg Hannon, Roido Manavaki, Tim D. Fryer, Luigi Aloj, Martin J. Graves, Fiona J. Gilbert, Marc R. Dweck, David E. Newby, Zahi A. Fayad, Gary Reynolds, Ann W. Morgan, Eric O. Aboagye, Anthony P. Davenport, Helle F. Jørgensen, Ziad Mallat, Martin R. Bennett, James E. Peters, James H.F. Rudd, Justin C. Mason, Jason M. Tarkin

**Affiliations:** a Section of Cardiorespiratory Medicine, University of Cambridge, Cambridge, United Kingdom; b Department of Radiology, Imperial College Healthcare National Health Service (NHS) Trust, London, United Kingdom; c Department of Radiology, Cambridge University Hospitals NHS Trust, Cambridge, United Kingdom; d Engineering and Physical Sciences Research Council Centre for Mathematical Imaging in Healthcare, University of Cambridge, Cambridge, United Kingdom; e Leeds Institute of Cardiovascular & Metabolic Medicine, University of Leeds, Leeds, United Kingdom; f Vascular Sciences, National Heart & Lung Institute, Imperial College London, London, United Kingdom; g Cancer Research UK Cambridge Institute, Cambridge, United Kingdom; h Department of Oncology, University College London NHS Trust, London, United Kingdom; i Department of Surgery & Cancer, Imperial College London, London, United Kingdom; j Department of Rheumatology, Cambridge University Hospitals NHS Trust, Cambridge, United Kingdom; k Department of Medicine, University of Cambridge, Cambridge, United Kingdom; l Department of Cardiology, Royal Papworth Hospital NHS Trust, Cambridge, United Kingdom; m Department of Histopathology, Cambridge University Hospitals NHS Trust, Cambridge, United Kingdom; n Department of Histopathology, Royal Papworth Hospital NHS Trust, Cambridge, United Kingdom; o Institute of Biomedical & Clinical Science, University of Exeter Medical School, Exeter, United Kingdom; p Department of Radiology, University of Cambridge, Cambridge, United Kingdom; q Department of Clinical Neurosciences, University of Cambridge, Cambridge, United Kingdom; r Centre for Cardiovascular Science, University of Edinburgh, Edinburgh, United Kingdom; s BioMedical Engineering & Imaging Institute, Icahn School of Medicine at Mount Sinai, New York, New York, USA; t Department of Rheumatology, University of Newcastle, Newcastle, United Kingdom; u Centre for Inflammatory Disease, Imperial College London, London, United Kingdom

**Keywords:** atherosclerosis, giant cell arteritis, inflammation, molecular imaging, somatostatin receptor, Takayasu arteritis, CRP, C-reactive protein, CT, computed tomography, FDG, fluorodeoxyglucose, GCA, giant cell arteritis, LVV, large vessel vasculitis, mds, most diseased segment, MI, myocardial infarction, MRI, magnetic resonance imaging, PET, positron emission tomography, PGA, Physician Global Assessment, RNAseq, RNA sequencing, ROI, region of interest, SEM, standard error of the mean, SMA, smooth muscle actin, SST_2_, somatostatin receptor 2, TAK, Takayasu arteritis, TBR, tissue-to-blood ratio

## Abstract

**Background:**

Assessing inflammatory disease activity in large vessel vasculitis (LVV) can be challenging by conventional measures.

**Objectives:**

We aimed to investigate somatostatin receptor 2 (SST_2_) as a novel inflammation-specific molecular imaging target in LVV.

**Methods:**

In a prospective, observational cohort study, in vivo arterial SST_2_ expression was assessed by positron emission tomography/magnetic resonance imaging (PET/MRI) using ^68^Ga-DOTATATE and ^18^F-FET-βAG-TOCA. Ex vivo mapping of the imaging target was performed using immunofluorescence microscopy; imaging mass cytometry; and bulk, single-cell, and single-nucleus RNA sequencing.

**Results:**

Sixty-one participants (LVV: n = 27; recent atherosclerotic myocardial infarction of ≤2 weeks: n = 25; control subjects with an oncologic indication for imaging: n = 9) were included. Index vessel SST_2_ maximum tissue-to-blood ratio was 61.8% (*P* < 0.0001) higher in active/grumbling LVV than inactive LVV and 34.6% (*P* = 0.0002) higher than myocardial infarction, with good diagnostic accuracy (area under the curve: ≥0.86; *P* < 0.001 for both). Arterial SST_2_ signal was not elevated in any of the control subjects. SST_2_ PET/MRI was generally consistent with ^18^F-fluorodeoxyglucose PET/computed tomography imaging in LVV patients with contemporaneous clinical scans but with very low background signal in the brain and heart, allowing for unimpeded assessment of nearby coronary, myocardial, and intracranial artery involvement. Clinically effective treatment for LVV was associated with a 0.49 ± 0.24 (standard error of the mean [SEM]) (*P* = 0.04; 22.3%) reduction in the SST_2_ maximum tissue-to-blood ratio after 9.3 ± 3.2 months. SST_2_ expression was localized to macrophages, pericytes, and perivascular adipocytes in vasculitis specimens, with specific receptor binding confirmed by autoradiography. *SSTR2*-expressing macrophages coexpressed proinflammatory markers.

**Conclusions:**

SST_2_ PET/MRI holds major promise for diagnosis and therapeutic monitoring in LVV. (PET Imaging of Giant Cell and Takayasu Arteritis [PITA], NCT04071691; Residual Inflammation and Plaque Progression Long-Term Evaluation [RIPPLE], NCT04073810)

Large vessel vasculitis (LVV) is a chronic relapsing and remitting systemic inflammatory disease that comprises giant cell arteritis (GCA) and Takayasu arteritis (TAK), which causes panarterial granulomatous infiltration of the aorta and its major branches. The initial clinical presentation of LVV is often confounded by nonspecific constitutional symptoms that can lead to diagnostic uncertainty. However, dangerous clinical sequelae such as acute visual loss and myocardial infarction (MI) can occur in GCA and TAK, respectively.

Although ^18^F-fluorodeoxyglucose (FDG) positron emission tomography (PET) imaging has traditionally been used to assess for the presence and severity of LVV, limitations including a high false-positive rate among LVV patients in clinical remission have been highlighted in clinical practice guidelines.^1^ ^18^F-FDG uptake related to chronic aortic inflammation in patients with atherosclerosis can also mimic LVV changes. Hence, although ^18^F-FDG PET is helpful for supporting an initial LVV diagnosis, it may be less useful for differentiating active arteritis from chronic vascular remodeling or for identifying residual disease activity after treatment. As a glucose analogue, avid physiologic ^18^F-FDG uptake in the brain and myocardium can also interfere with reliable assessment of temporal arteritis in GCA and coronary artery involvement in TAK.

We previously demonstrated the ability of somatostatin receptor 2 (SST_2_) PET/computed tomography (CT) to detect active inflammation in atherosclerosis using ^68^Ga-DOTATATE.^2^ *SSTR2* is expressed by inflammatory macrophages activated in vitro, and SST_2_ staining colocalizes with CD68^+^ macrophages within inflamed carotid atherosclerotic plaques. Of the somatostatin receptor PET tracers used for clinical neuroendocrine tumor imaging, ^68^Ga-DOTATATE has the highest binding affinity for SST_2_. A novel ^18^F click-labeled octreotide radioligand called ^18^F-FET-βAG-TOCA has also shown high SST_2_ binding affinity and favorable tracer kinetics.^3^ In this proof-of-concept study, we tested the hypothesis that SST_2_ could be a useful imaging target for the diagnosis and therapeutic monitoring of LVV using PET/magnetic resonance imaging (MRI) with ^68^Ga-DOTATATE and ^18^F-FET-βAG-TOCA.

## Methods

Research was conducted with the approval of local research ethics committees (19/EE/0043; 05/Q1108/28; 16/NE/0319) in accordance with the Declaration of Helsinki. The retention, storage, and use of tissue sections and blood samples were subject to the UK Human Tissue Act 2004. See the Supplemental Appendix for an extended Methods section.

### Clinical study

In this prospective observational cohort study (NCT04071691), participants with LVV based on American College of Rheumatology diagnostic criteria were enrolled from 2 hospitals in the United Kingdom (Cambridge University Hospitals National Health Service Trust and Imperial College Healthcare National Health Service Trust). Patients were managed following standard clinical practice guidelines for LVV. Clinical LVV activity status was graded independent of the study findings by 3 experienced rheumatologists as “active” (new diagnosis or acute flare), “grumbling” (low-grade residual arteritis), or “inactive” (disease in remission), according to the Physician Global Assessment (PGA). The PGA is an overall assessment of LVV activity based on clinical symptoms, signs, and inflammatory blood markers. The Indian Takayasu Clinical Activity Score was additionally used for patients with TAK (Supplemental Figure 1). Participants with atherosclerotic MI within 2 weeks enrolled in a parallel PET/MRI study that is part of the same larger project (NCT04073810), and control subjects from a previous oncology study^3^ were also included. Venous blood was collected at the time of imaging.

### Imaging

SST_2_ imaging was performed using ^68^Ga-DOTATATE (in Cambridge) or ^18^F-FET-βAG-TOCA (in London) on an integrated PET/MRI scanner (SIGNA, GE Healthcare) with a target injected activity of 250 MBq, 50-min circulation time, and 30-min acquisition per bed position. Two bed positions were used to image the thoracic aorta and, subsequently, the head and neck vessels in patients with LVV, and a single bed position focused on the heart/aorta was acquired in MI patients. PET images were reconstructed using iterative time of flight (Q.Clear β = 350) and a free-breathing 2-point Dixon MRI sequence for attenuation correction. The 3 Tesla MRI included breath-held proton-density weighted, blood-suppressed single-shot fast-spin echo aortic imaging, 3D carotid time-of-flight magnetic resonance angiography, T_1_-weighted 3D fast spin echo with fat suppressed, and gadolinium contrast-enhanced magnetic resonance angiography. Multibed whole-body ^18^F-FET-βAG-TOCA PET/CT was performed as part of a separate oncology trial using a PET/CT scanner (Biograph, Siemens) with ordered-subsets expectation maximization reconstruction, as previously described.^3^

### Image analysis

Arterial radioactivity concentration measured as the standardized uptake value was derived from 2D regions of interest (ROIs) drawn around the outer vessel boundaries of the thoracic aorta, proximal aortic arch vessels, and carotid and vertebral arteries on consecutive coregistered PET/MRI slices, with readers blinded to clinical details using open-source medical imaging software (Horos, version 3.3.6), and normalized by blood pool activity to generate mean and most diseased segment (mds) maximum tissue-to-blood ratios (TBR_max_) values.^4^ The mds was defined as the highest arterial TBR_max_ slice, averaged with contiguous slices above and below. The index vessel was the artery with the highest mean TBR_max_. Intraobserver and interobserver repeatability of these methods has previously been demonstrated using ^68^Ga-DOTATATE.^2^

### RNA sequencing

Bulk RNA-sequencing (RNAseq) data are from the UKGCA Consortium Study (NCT04102930), which is linked to the National Institute for Health Research Rare Diseases BioResource. Single-cell and single-nucleus RNAseq were performed using Chromium (10x Genomics).

### Histology and autoradiography

Arterial specimens were analyzed using methods for immunofluorescence microscopy as previously described,^5^ with primary antibodies for SST_2_ and CD68. ^68^Ga-DOTATATE autoradiography was conducted using established methods.^2^ Imaging mass cytometry was performed in temporal artery sections using fluorescein isothiocyanate–conjugated SST_2_ and a panel of 10 metal-conjugated antibodies.

### Statistical analysis

Statistical analyses were performed using R version 4.0.2 (R Core Team) and Prism version 9.1.0 (GraphPad). Data are expressed as median (IQR) or mean ± SD, as appropriate. Group comparisons were made using the Kruskal-Wallis test and Wilcoxon matched-pairs signed rank test. Receiver-operating characteristic (ROC) analysis was used to assess diagnostic accuracy and identify optimal TBR thresholds based on the Youden index. Linear mixed-effects models were used to account for hierarchical data structure and multiple observations within patients, with patient and vessel included as random effects and tracer as a fixed effect. A random-effects regression model was used to assess ΔTBR at follow-up compared with baseline. Results of the regression models are reported as effect size ± SEM or absolute change (95% CI) for log-transformed data, as well as the percent difference between groups. Potential confounding factors associated with mean TBR_max_ (*P* ≤ 0.10) in univariable linear mixed-effects models were included in multivariable sensitivity analyses. A 2-sided *P* value of <0.05 was considered significant.

## Results

**Figure 1:**
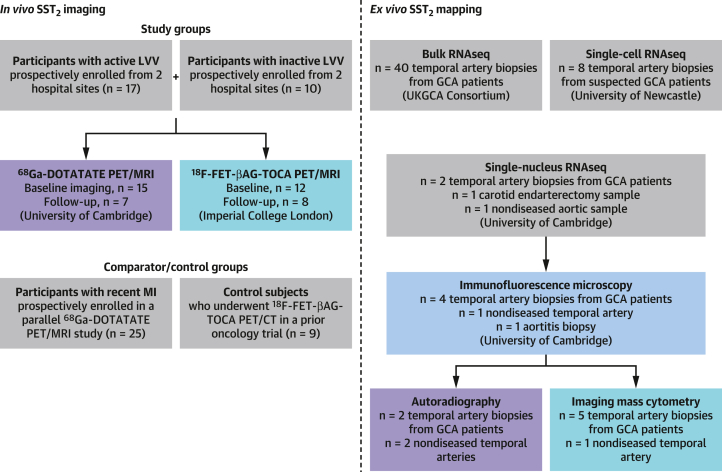
Patient Cohorts and Tissue Samples Flowchart summarizing the patient cohorts and arterial samples included in the study. Participants with active and inactive LVV were enrolled from 2 hospital sites. PET/MRI scans from patients with LVV were compared to those of patients with recent MI enrolled in a parallel study and control subjects from a prior oncology trial. Sources of arterial samples and RNAseq data from patients with GCA, carotid atherosclerosis, and nondiseased control arteries used to evaluate target expression of somatostatin receptor 2 in LVV are as shown. DOTATATE = DOTA-(Tyr3)-octreotate; FET = fluoroethyltriazole; GCA = giant cell arteritis; LVV = large vessel vasculitis; MI = myocardial infarction; MRI = magnetic resonance imaging; PET = positron emission tomography; RNAseq = RNA sequencing.

Sixty-one participants (LVV: n = 27; recent MI: n = 25; control subjects: n = 9) (Figure 1) were included. Baseline clinical data are summarized in Table 1. Patients with MI were imaged a median 8 days (IQR: 6-8 days) after MI and had a median troponin I level of 25,000 ng/L (IQR: 3,491-25,000) at the time of infarct. All but 2 patients with MI underwent percutaneous coronary intervention to the culprit lesion. The mean injected activities and uptake times were 222 ± 20 MBq and 53 ± 4 minutes for ^68^Ga-DOTATATE and 212 ± 35 MBq and 56 ± 6 minutes for ^18^F-FET-βAG-TOCA at baseline.

**Table 1:** Baseline Clinical Characteristics

	LVV (n = 27)	Myocardial Infarction (n = 25)	Control Subjects (n = 9)
Age, y	63 (49-68)	61 (54-65)	56 (47-69)
Female	21 (78)	5 (20)	6 (67)
BMI, kg/m^2^	27.9 (25.5-30.3)	28.7 (26.0-32.9)	26.3 (24.0-30.4)
LVV diagnosis			
GCA	13 (48)	n/a	n/a
Takayasu arteritis	13^a^ (48)	n/a	n/a
Unspecified LVV	1^b^ (4)	n/a	n/a
LVV clinical disease activity status			
Active	11 (41)	n/a	n/a
Grumbling	6 (22)	n/a	n/a
Inactive	10 (37)	n/a	n/a
Medical history			
Hypertension	14 (52)	12 (48)	3 (33)
Hypercholesterolemia	9 (33)	13 (52)	0 (0)
Diabetes mellitus	3 (11)	4 (16)	1 (11)
Chronic kidney disease	1 (4)	0 (0)	0 (0)
Atrial fibrillation	2 (7)	2 (8)	0 (0)
Stable angina	5 (19)	1 (4)	0 (0)
Myocardial infarction	0 (0)	25 (100)	1 (11)
Coronary artery bypass grafting surgery	1 (4)	0 (0)	0 (0)
Stroke or transient ischemic attack	1 (4)	0 (0)	0 (0)
Peripheral vascular disease	2 (7)	0 (0)	0 (0)
Rheumatoid arthritis	3 (11)	0 (0)	0 (0)
Psoriasis	1 (4)	0 (0)	0 (0)
Systemic lupus erythematosus	0	1 (4)	0
Current or past smoking habit	8 (30)	15 (60)	—
Family history of early coronary heart disease	2 (7)	9 (26)	—
Baseline immunosuppression			
Corticosteroid	17 (63)	1 (4)	0 (0)
Methotrexate	9 (33)	0 (0)	0 (0)
Azathioprine	1 (4)	0 (0)	0 (0)
Cyclophosphamide	1 (4)	0 (0)	0 (0)
Mycophenolate mofetil	2 (7)	0 (0)	0 (0)
Tocilizumab	4 (15)	0 (0)	0 (0)
Statin therapy	11 (41)	25 (100)	1 (11)
Antihypertensive therapy	13 (48)	24 (96)	7 (78)
Baseline blood tests^c^			
High-sensitivity C-reactive protein, mg/L (NR: <3.0)	4.24 (0.76-19.60)	4.93 (3.01-11.43)	—
High-sensitivity troponin I, n/L (NR: <40)	4.20 (2.00-6.60)	206 (13.5-1,132)	—
Erythrocyte sedimentation rate, mm/h (NR: <35)	16 (6-41)	—	—
Interleukin 6, pg/mL	25.08 (10.33-71.55)	57.32 (8.01-121.2)	—
Pentraxin-3, ng/mL	2.16 (1.13-3.80)	2.80 (1.10-9.13)	—
Total cholesterol, mmol/L	4.50 (3.60-5.30)	3.60 (3.15-4.30)	—
Mean triglycerides, mmol/L	1.30 (1.06-1.94)	1.25(1.00-1.83)	—
Mean HDL cholesterol, mmol/L	1.27 (1.05-1.79)	0.94 (0.81-1.09)	—
Mean LDL cholesterol, mmol/L	2.23 (1.77-3.04)	1.99 (1.51-2.47)	—

Values are median (IQR) or n (%).

BMI = body mass index; GCA = giant cell arteritis; HDL = high-density lipoprotein; LDL = low-density lipoprotein; LVV = large vessel vasculitis; n/a = not applicable; NR = normal range.

a 1 patient with active Takayasu arteritis also had a non–ST-segment elevation myocardial infarction secondary to atherosclerotic plaque rupture 5 days before baseline positron emission tomography/magnetic resonance imaging.

b The patient with unspecified LVV was a 55-year-old man with history of Sjögren disease and stroke in whom vasculitis was diagnosed based on constitutional symptoms, raised C-reactive protein level, and classical imaging findings.

c Blood results shown are from the day of baseline positron emission tomography/magnetic resonance imaging.

### LVV clinical disease activity

**Figure 2:**
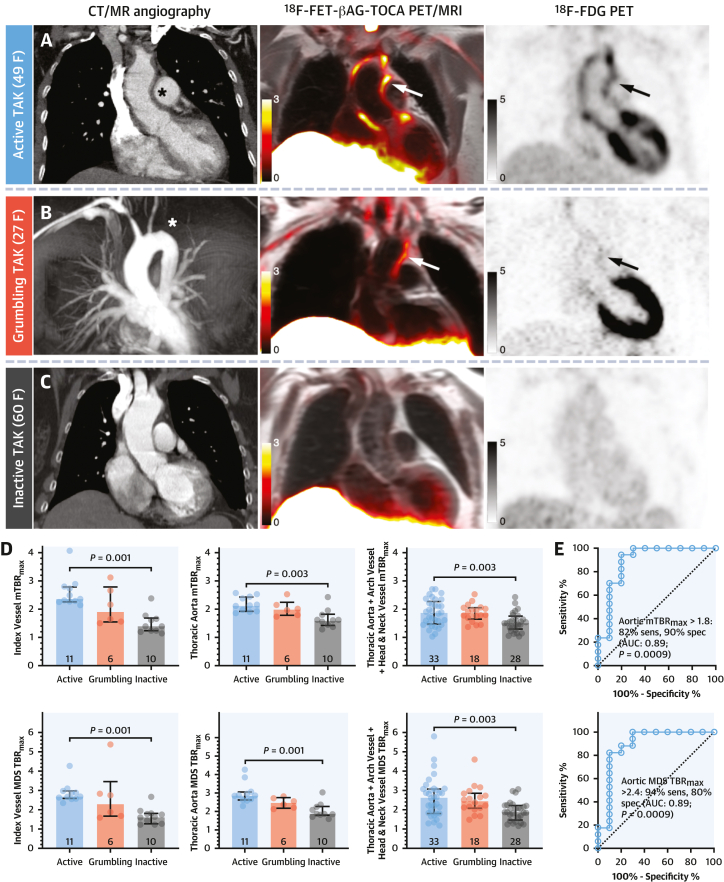
Clinical LVV Activity Aortic SST_2_ PET/MRI signals **(white arrows)** in TAK patients grouped by clinical disease activity: **(A)** active disease (treatment naive at baseline imaging) with arterial thickening **(asterisk)**, **(B)** grumbling disease with left subclavian occlusion (asterisk) and brachiocephalic stenosis, and **(C)** inactive disease with no aortic thickening. Contemporaneous ^18^F-fluorodeoxyglucose (FDG) PET images **(black arrows)**. **(D)** Quantitative comparisons of mean and most diseased segment SST_2_ PET TBR_max_. **(E)** Receiver-operating characteristic analyses of SST_2_ PET mean TBR_max_ and mdsTBR_max_ for differentiating active/grumbling from inactive LVV. **Image scale bars** indicate standardized uptake value; **error bars inD** indicate median (IQR). ^18^F-FDG = ^18^F-fluorodeoxyglucose; AUC = area under the curve; CT = computed tomography; F = female; m = mean; mds = most diseased segment; MR = magnetic resonance; sens = sensitivity; spec = specificity; SST_2_ = somatostatin receptor 2; TAK = Takayasu arteritis; TBR_max_ = maximum tissue-to-blood ratio; other abbreviations as in Figure 1.

To test the accuracy of SST_2_ PET for inflammatory disease activity in LVV, 3,828 ROIs were analyzed from 27 baseline PET/MRI scans. Both mean TBR_max_ and mdsTBR_max_ differed among patients with clinically active, grumbling, and inactive LVV for the index vessels, thoracic aorta, and all vessels combined (*P* < 0.005 for all) (Figures 2A to 2D, Supplemental Table 1). Data for the 2 tracers were pooled because there was comparable image quality and ability to distinguish active/grumbling LVV from inactive LVV (Supplemental Figure 2). Aortic mean TBR_max_ of >1.8 and mdsTBR_max_ of >2.4 demonstrated the best diagnostic accuracy for differentiating active/grumbling LVV from inactive LVV (area under curve: 0.89; *P* = 0.0009 for both) (Figure 2E). In the linear mixed-effects model, individual baseline TBR_max_ values for index vessels were 61.8% (95% CI: 31.5%-99.0%; *P* < 0.0001) higher in active/grumbling LVV than inactive LVV (Supplemental Table 2). For the thoracic aorta, the difference was 26.6% (95% CI: 12.6%-42.3%; *P* < 0.0001), and for all vessels, it was 11.6% (95% CI: 1.0%-23.4%; *P* = 0.03).

### LVV vs atherosclerotic inflammation

**Figure 3:**
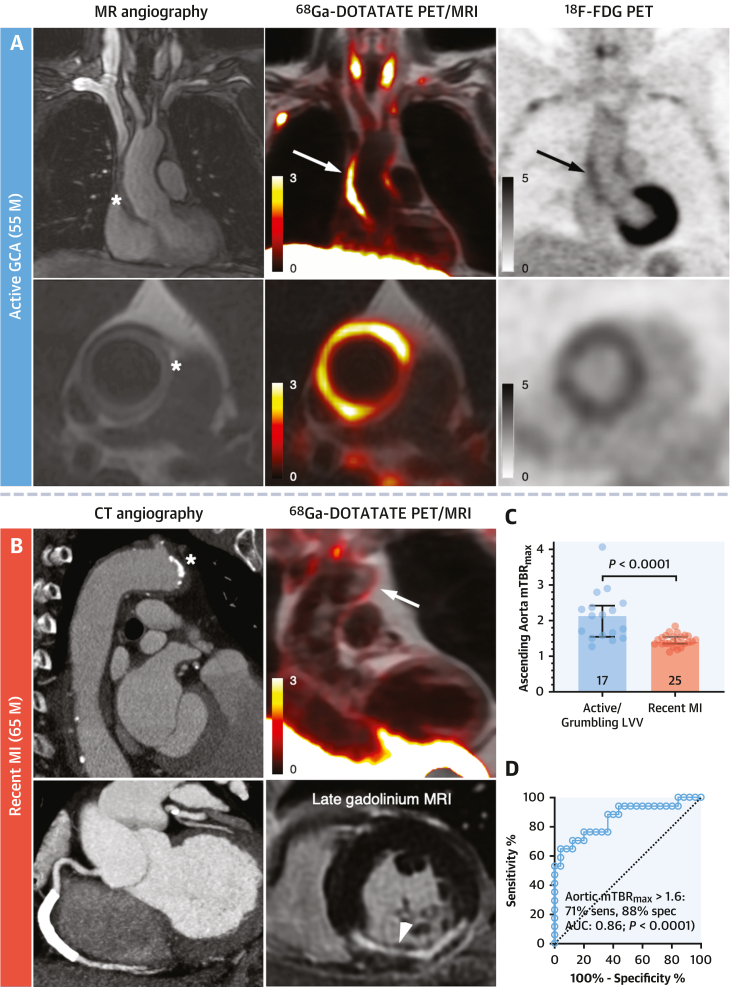
Vasculitis vs Atherosclerotic Inflammation Aortic SST_2_ PET/MRI signals **(white arrows)** in patients with **(A)** active GCA (treatment naive at baseline imaging) and aortic thickening **(asterisk)** and **(B)** recent MI with aortic atherosclerosis **(asterisk)** and inferior infarction (**arrowhead**; note the infarct-related myocardial PET uptake). Contemporaneous ^18^F-FDG PET images in **A** show aortic uptake **(black arrows)**. **(C)** Quantitative comparison of aortic mean SST_2_ PET TBR_max_. **(D)** Receiver-operating characteristic analysis of SST_2_ PET mean TBR_max_ for differentiating active/grumbling from inactive LVV. **Image scale bars** indicate SUV; **error bars** in **C** indicate median (IQR). M = male; other abbreviations as in Figures 1 and 2.

To determine the accuracy of SST_2_ PET for discriminating vasculitis from aortic inflammation caused by atherosclerosis, 153 ROIs were analyzed from the ascending aortas of 17 patients with active/grumbling LVV and 182 ROIs in the ascending aortas of 25 patients with recent MI. Focal ^68^Ga-DOTATATE signals relating to aortic atherosclerosis in MI patients were of much lower intensity than in LVV and appeared patchy rather than circumferential. Aortic SST_2_ PET mean TBR_max_ was higher in patients with active/grumbling LVV than recent MI (*P* < 0.0001) (Figures 3A to 3C). When including only MI patients with aortic atherosclerosis visible on CT angiography (n = 15 [60%]) and also when comparing descending aorta mean TBR_max_, the difference remained (*P* < 0.005 for both) (Supplemental Figure 3). Aortic mean TBR_max_ of >1.6 had the best diagnostic accuracy for differentiating patients with active/grumbling LVV from recent MI (area under the curve: 0.86; *P* < 0.0001) (Figure 3D). In the linear mixed-effects model, individual TBR_max_ values were 34.6% (95% CI: 15.1%-57.6%; *P* = 0.0002) higher in active/grumbling LVV than recent MI.

### Control subjects

Unlike patients with active vasculitis, none of the control subjects had aortic ^18^F-FET-βAG-TOCA increased above the background (Supplemental Figure 4).

### ^18^F-FDG PET imaging

**Figure 4:**
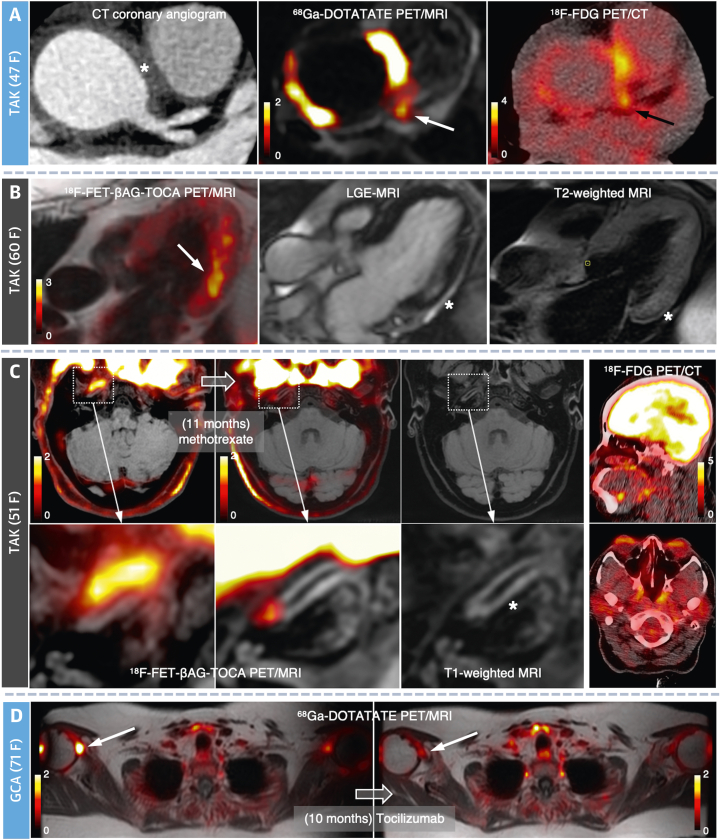
Varied Patterns of LVV Involvement SST_2_ PET/MRI signals **(white arrows)** in **(A)** the aortic root and left main coronary artery with adjacent periaortic thickening **(asterisk)** and ^18^F-FDG PET uptake **(black arrow)** in a patient with active TAK, **(B)** the basal inferolateral left ventricular myocardium in a patient with inactive TAK and subclinical myocarditis confirmed by mid-wall late gadolinium enhancement **(asterisk)** and increased T_2_-edema signal (asterisk), **(C)** the thickened intracranial portion of the right internal carotid artery **(asterisk)** in a patient with TAK and previous stroke in whom ^18^F-FDG failed to detect this abnormality, and **(D)** the right glenohumeral joint in a patient with GCA and polymyalgia rheumatica symptoms. Note that there is a reduction in PET signal intensity following immunosuppressive treatment in **C** and **D**. **Image scale bars** indicate SUV. LGE = late gadolinium enhancement; other abbreviations as in Figures 1 and 2.

**Figure 5:**
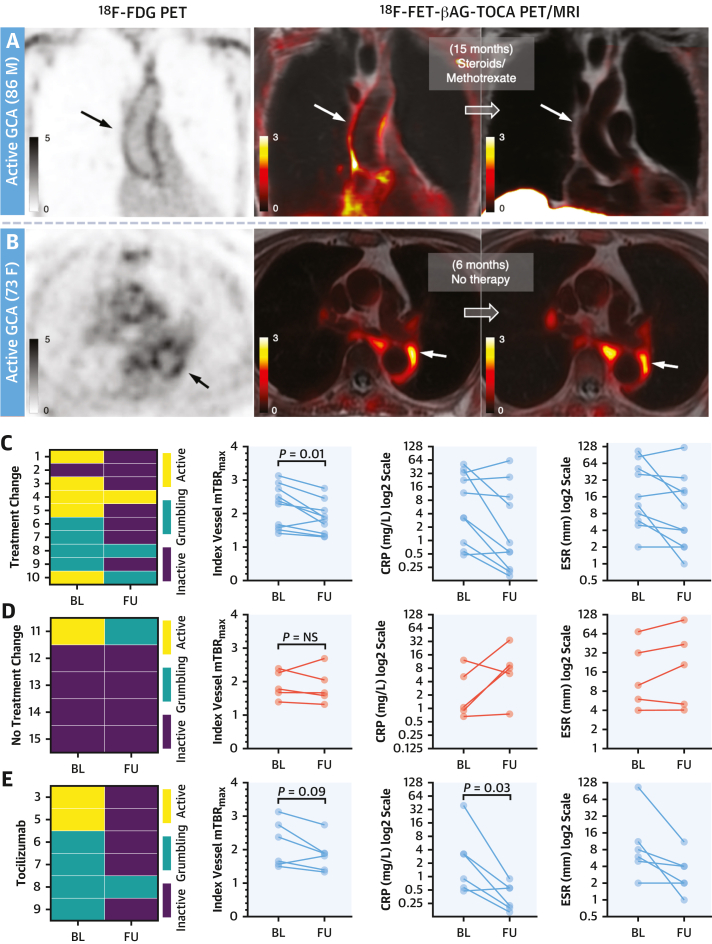
Repeat Imaging Baseline and follow-up images from patients with **(A)** newly diagnosed active GCA (treatment naive at baseline imaging) and **(B)** active GCA who, of their own volition, remained off treatment during the study because of side effects from prednisolone and methotrexate (subsequently well controlled with tocilizumab). SST_2_ PET/MRI shows resolution in aortic inflammation **(white arrows)** after treatment and no change with lack of treatment. Contemporaneous (pretreatment) ^18^F-FDG PET images showing similar aortic uptake **(black arrows)** to SST_2_ PET. **(C to E)** Graphs showing changes in baseline vs follow-up clinical disease activity grading, index vessel mean SST_2_ TBR_max_, CRP, and ESR in **(C)** patients with any escalation in treatment, **(D)** those with no treatment change, and **(E)** those who received tocilizumab as part of their therapy regime. Note that the patients in **E** are a subset of those in **C**. Further clinical data for each patient who underwent repeat imaging are provided in Supplemental Table 4. **Image scale bars** indicate SUV. BL = baseline; CRP = C-reactive protein; ESR = erythrocyte sedimentation rate; FU = follow-up; other abbreviations as in Figures 1, 2, and 3.

^18^F-FDG PET/CT imaging was performed for clinical care and was not part of the research protocol of this initial study. However, in cases where contemporaneous clinical ^18^F-FDG imaging was performed within 1 year of the baseline imaging (n = 10; median scan-scan interval: 127 days [IQR: 38-245 days]) and in another patient where the interscan interval was longer (15 months), where both the treatment and PGA score remained unchanged, there was remarkably good agreement observed for SST_2_ PET/MRI and ^18^F-FDG PET/CT in 9 (82%) scans (Figures 2A to 2C, 3A, 4A, 5A, and 5B, Supplemental Figure 5). In 2 of these patients, ^18^F-FDG PET showed no arterial uptake despite a clinical suspicion of active disease; however, SST_2_ revealed increased signal in the affected vertebral (Supplemental Figure 5B) and intracranial (Figure 4C) arteries.

### Patterns of LVV involvement

Unlike ^18^F-FDG, background SST_2_ PET activity was very low in the brain and healthy myocardium, allowing the potential for unimpeded assessment of nearby vessels. None of the patients in the study had symptoms of active temporal arteritis at the time of PET/MRI because of the need for urgent treatment. However, SST_2_ PET identified LVV disease activity in patients with coronary arteritis, subclinical myocarditis, and intracranial vasculitis (Figures 4A to 4C). Other patients with GCA and polymyalgia rheumatica symptoms had glenohumeral joint tracer uptake (Figure 4D).

### Therapeutic monitoring

Fifteen patients with LVV underwent repeat SST_2_ PET/MRI (scan-scan interval median: 9.6 months [IQR: 5.5-11.4 months]; mean injected activities and uptake times at follow-up: ^68^Ga-DOTATATE: 162 ± 44 MBq and 59 ± 8 minutes; ^18^F-FET-βAG-TOCA: 174 ± 45 MBq and 67 ± 4 minutes). Further details are in Supplemental Table 3. Although it was intended to repeat imaging for all patients after 6 months, some scans were delayed during the COVID-19 pandemic, and 12 patients declined to attend for the second scan because they remained in isolation when public restrictions were lifted. Ten of the 15 LVV patients who did undergo repeat imaging had newly initiated or escalated treatment after their baseline scan, which was associated with clinical improvement based on the PGA score in 8 of these patients.

Patients with newly initiated or escalated treatment (n = 10) had lower SST_2_ PET mean TBR_max_ in the index vessel at follow-up than baseline (*P* = 0.01) (Figures 5A and 5C). When comparing individual TBR_max_ values in a linear random-effects model, clinically effective treatment for LVV (defined as any improvement in PGA score) was associated with a 0.49 ± 0.24 (SEM) (*P* = 0.04; 22.3%) reduction in index vessels, 0.32 ± 0.09 (SEM) (*P* = 0.0003; 14.5%) reduction in the thoracic aorta, and 0.39 ± 0.17 (SEM) (*P* = 0.02; 18.4%) reduction across all vessels.

### Effect of interleukin-6 receptor blocking

In 6 patients treated with tocilizumab (a monoclonal antibody against the interleukin-6 receptor) as per standard clinical dosing for relapsing or refractory LVV, there was a trend toward reduction in the index vessel mean TBR_max_ at follow-up (*P* = 0.09) (Figure 5E). In patients whose PGA score was improved by tocilizumab (n = 5), there was a reduction in TBR_max_ at follow-up using a linear random-effects model for the thoracic aorta (–0.29 ± 0.11 [SEM]; *P* = 0.009; 13.8%) and across all vessels (–0.36 ± 0.18 [SEM]; *P* = 0.04; 17.7%) but no change in the index vessels. In the 1 patient whose clinical symptoms failed to respond to tocilizumab, arterial SST_2_ PET signal remained elevated at follow-up despite a C-reactive protein (CRP) level of <1.0 mg/L.

### PET/MRI repeatability

Scan-scan repeatability was also evaluated. Importantly, there was no difference in index vessel mean TBR_max_ in the 5 patients with LVV whose treatment remained unchanged (scan-scan interval: 9.2 ± 3.8 months) (Figures 5B and 5D). The single measure intraclass correlation coefficient, using a 2-way mixed-effects model with absolute agreement for index vessel mean TBR_max_ values from baseline and follow-up scans in LVV patients with inactive disease and no change in treatment (n = 4 patients), was 0.86 (95% CI: 0.04-0.99). The mean bias of individual TBR_max_ values (n = 47 ROIs) between scans for these patients was 0.16 ± 0.32 on Bland-Altman analysis (Supplemental Figure 6).

### Systemic inflammatory markers

In contrast to the PET imaging findings, there were no differences in any of the blood inflammatory markers tested between patients with active/grumbling LVV and inactive LVV. There were also no differences in these markers between patients with active/grumbling LVV and recent MI. However, after excluding the 4 patients whose baseline treatment included tocilizumab, there was a difference in CRP between active/grumbling LVV vs inactive LVV (*P* = 0.02) and a trend toward difference in active/grumbling LVV vs recent MI (*P* = 0.08) (Supplemental Figures 7A and 7B). The effects of new or escalated LVV treatments on CRP and other inflammatory markers were varied, with no difference at follow-up compared to baseline aside for tocilizumab (Figures 5C and 5E). Although there was a moderate correlation between aortic mdsTBR_max_ and CRP in patients with LVV (*r* = 0.44; 95% CI: 0.06-0.71; *P* = 0.02) (Supplemental Figure 7C), there were no other associations between PET activity and inflammatory markers. However, SST_2_ PET was strongly correlated with the Indian Takayasu Clinical Activity Score–CRP score in patients with TAK (mean TBR_max_: *r* = 0.82; 95% CI: 0.46-0.95; *P* = 0.001; mdsTBR_max_; *r* = 0.75; 95% CI: 0.29-0.93; *P* = 0.006) (Supplemental Figure 7D).

### Comparison with MRI

Aortic thickening assessed by MRI (>2.2 mm) occurred in 70% (19 of 27) of LVV patients. Aortic mean TBR_max_ and mdsTBR_max_ were greater in LVV patients with aortic thickening than those without (*P* < 0.005 for both) (Supplemental Figure 8A). Aortic mean TBR_max_ was also correlated with maximum wall thickness (*r* = 0.68; *P* = 0.002) (Supplemental Figure 8B) in these patients.

### Multivariable regression analysis

After adjustment for potential confounders with *P* ≤ 0.10 in univariable analysis (Supplemental Table 4), the difference in TBR_max_ between active/grumbling LVV and inactive LVV became more pronounced across all vessels (18%; 95% CI: 5.8-31.5; *P* = 0.003). For the index vessel and aorta, the differences remained unchanged.

### Ex vivo mapping of *SSTR2*

**Figure 6:**
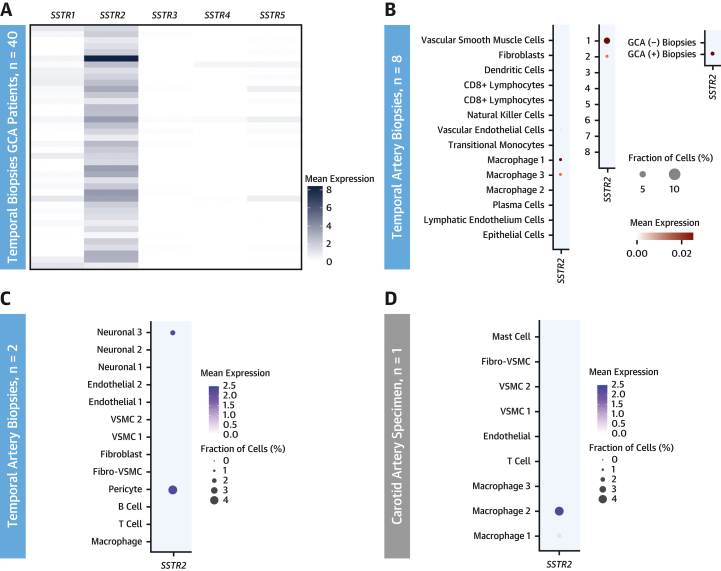
RNA Sequencing Plots of **(A)** bulk, **(B)** single-cell, and **(C, D)** single-nucleus RNAseq data from temporal arteries and a carotid atherosclerotic specimen, showing *SSTR2* expression localized to populations of macrophages and pericytes. Gene-level expression data displayed as normalized counts per million in **A** confirm that *SSTR2* is the dominant somatostatin receptor subtype expressed in temporal arteritis. In **B**, labels 1 to 8 indicate data from individual patients. GCA = giant cell arteritis; VSMC = vascular smooth muscle cell.

Bulk RNAseq data from temporal artery biopsy samples of GCA patients diagnosed by American College of Rheumatology criteria (n = 40; mean age: 75 years; range: 60-92 years; 24 [60%] female) showed increased expression of *SSTR2* compared to other somatostatin receptors (Figure 6A). There was no association between *SSTR2* expression and steroid duration (median: 6 days; range: 0-16 days). However, *SSTR2* was correlated with *CD68* at both the gene (*r* = 0.34, *P* = 0.03) and transcript levels (range: *r* = 0.33-0.40, *P* < 0.05) (Supplemental Table 5).

Single-cell RNAseq data (n = 8; mean age: 72 years; range: 64-84 years; 5 [63%] female; median steroid duration: 10 days; range: 4-13 days) localized *SSTR2* expression to macrophages in temporal artery biopsy specimens from 2 of 5 patients with confirmed GCA based on clinical and histologic criteria (Figure 6B). These 2 patients had positive ultrasound findings for temporal arteritis and the highest CRP levels at presentation of the cohort. *SSTR2* was not detected in any of the 3 samples without clinical or histologic features of GCA in the single-cell RNAseq data set.

To further corroborate these findings, single-nucleus RNAseq was performed in temporal artery biopsy samples from patients with histologically proven GCA (n = 2) (Figure 6C) and a carotid endarterectomy specimen (n = 1) (Figure 6D). Patient details for these and other specimens are in Supplemental Table 6. Uniform Manifold Approximation and Projections and numbers of nuclei for cell clusters are shown in Supplemental Figure 9 and Supplemental Table 7. Macrophages again emerged as the dominant *SSTR2*-expressing cell type in the carotid atherosclerotic plaque, and pericytes were also identified in temporal biopsy samples. *SSTR2* expression was not detected by single-nucleus RNAseq in a healthy aortic specimen (n = 1; not shown).

Cell clusters in the single-cell and single-nucleus experiments were not directly comparable. However, *SSTR2*-expressing macrophages identified in temporal arteries expressed proinflammatory markers (S100A8 and S100A9) (Supplemental Figure 10A). *SSTR2*-expressing macrophages in the carotid artery expressed markers of resident and/or alternatively activated macrophages (MERTK, SOD2, LGALS3) but also CXCL3, which is an inflammatory cytokine (Supplemental Figures 10C and 10D). Pericytes were not distinguished as a distinct population in the single-cell RNAseq data set.

### SST_2_ receptor immunostaining

**Figure 7:**
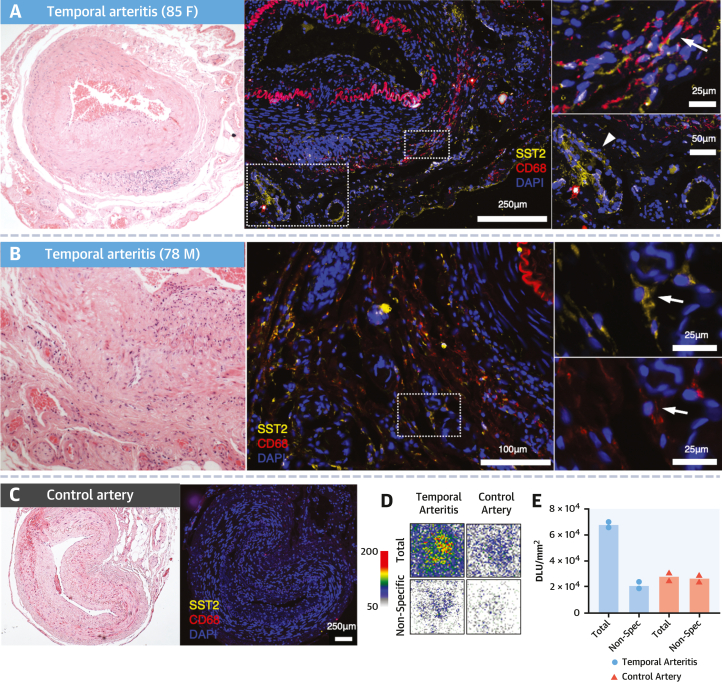
SST_2_ Staining and Autoradiography in Temporal Arteritis **(A, B)** Histologic images from patients with temporal arteritis showing immunofluorescence SST_2_ costaining in CD68^+^ macrophages **(arrow)**, as well as cells with the morphologic appearance of pericytes **(arrowhead)**, with corresponding hematoxylin and eosin slides. **(C)** Control artery shows no SST_2_ staining. **(D)** Autoradiographic images and **(E)** quantitative autoradiographic data confirm higher specific binding of ^68^Ga-DOTATATE to SST_2_ receptors in temporal arteritis specimens than control arteries. For **A** and **B**, patients had received prednisolone 40 mg for 20 days and 10 days, respectively, before undergoing temporal artery biopsy. Abbreviations as in Figures 1, 2, and 3.

Immunofluorescence microscopy was performed to verify the expression and cellular distribution of SST_2_ receptors within sections of the same artery biopsy specimens analyzed for single-nucleus RNAseq (n = 2), as well as additional temporal artery samples (GCA: n = 2; control artery with no abnormality: n = 1) and an aortic LVV specimen (n = 1). Histologic findings were consistent with RNAseq data. There was specific costaining of SST_2_ and the pan-macrophage marker CD68 within inflamed regions of temporal arteries (Figures 7A and 7B) and aortic tissue (Supplemental Figure 11), as well as SST_2_ staining of cells surrounding microvessels in the adventitia with the morphologic appearance of pericytes (confirmed by α-smooth muscle actin [αSMA]/neuron-glial antigen 2 staining using imaging mass cytometry). There was minimal SST_2_ staining in the control artery (Figure 7C).

### Autoradiography

Autoradiographic binding of ^68^Ga-DOTATATE to SST_2_ receptors was confirmed in temporal artery sections of patients with active GCA (n = 2) and compared with control arteries from patients without vasculitis (n = 2). There was higher specific binding of ^68^Ga-DOTATATE in the LVV specimens than nondiseased control arteries and very low nonspecific binding when blocked with an unlabeled cold competing compound (Figure 7D). Quantification of autoradiographic signal further confirmed these findings (Figure 7E).

### Imaging mass cytometry

**Figure 8:**
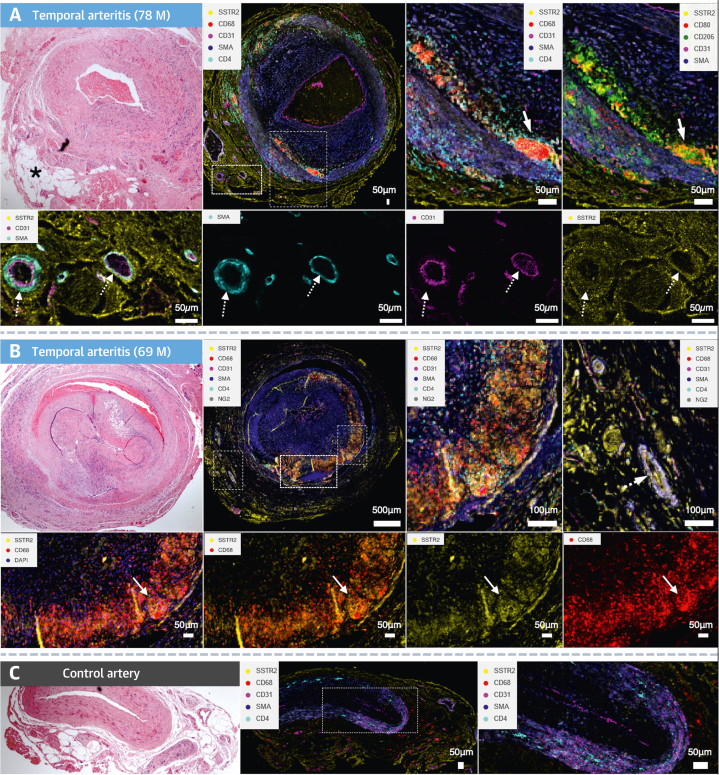
Localization of SST_2_ in Temporal Arteritis Using Imaging Mass Cytometry **(A, B)** Histologic images from patients with temporal arteritis performed using imaging mass cytometry showing costaining of SST_2_ with clusters of macrophages (CD68^+^/CD80^+^/CD206^+^, arrows) and pericytes (αSMA^+^/NG2^+^, dashed arrows) around neovessels in the periadventitia as well as periadventitial cells with adipocyte morphology **(asterisk)**, with related hematoxylin and eosin slides shown. **(C)** In contrast, there is minimal SST_2_ staining in the control artery and perivascular tissue. Sections in **A** are from the same patient as in Figure 6B. M = male.

Imaging mass cytometry was used to further delineate patterns of cell-type–specific SST_2_ expression in temporal artery biopsy samples (n = 6). Within CD68^+^ regions, SST_2_ appeared more closely colocalized with the inflammatory macrophage marker CD80 than with CD206, although there was overlap with both markers (Figure 8A). SST_2_ staining did not colocalize with CD31 in the endothelium or αSMA in the media of the main vessels. There was also no overlap with CD3^+^ or CD4^+^ T lymphocytes. However, cellular localization of SST_2_ did occur with pericytes identified by neuron-glial antigen 2^+^ and αSMA around neovessels in the adventitia (Figures 8A and 8B), as well as cells with the morphologic appearance of perivascular adipocytes. SST_2_ staining was not detected in the control artery and was low in perivascular tissue (Figure 8C).

## Discussion

Here, we show for the first time, to our knowledge, that SST_2_ receptors are expressed by inflammatory macrophages, as well as pericytes and perivascular adipocytes, within inflamed arteries of patients with LVV and can be detected using PET/MRI (Central Illustration). By repurposing existing PET tracers for this novel application, we describe a method that has the potential to be rapidly incorporated into clinical practice. Moreover, we tested both ^68^Ga-DOTATATE and a newer ^18^F-octreotide analogue that could further accelerate clinical translation by allowing easier transportation to hospitals without onsite cyclotron or gallium-68 generator facilities.

**Central Illustration:**
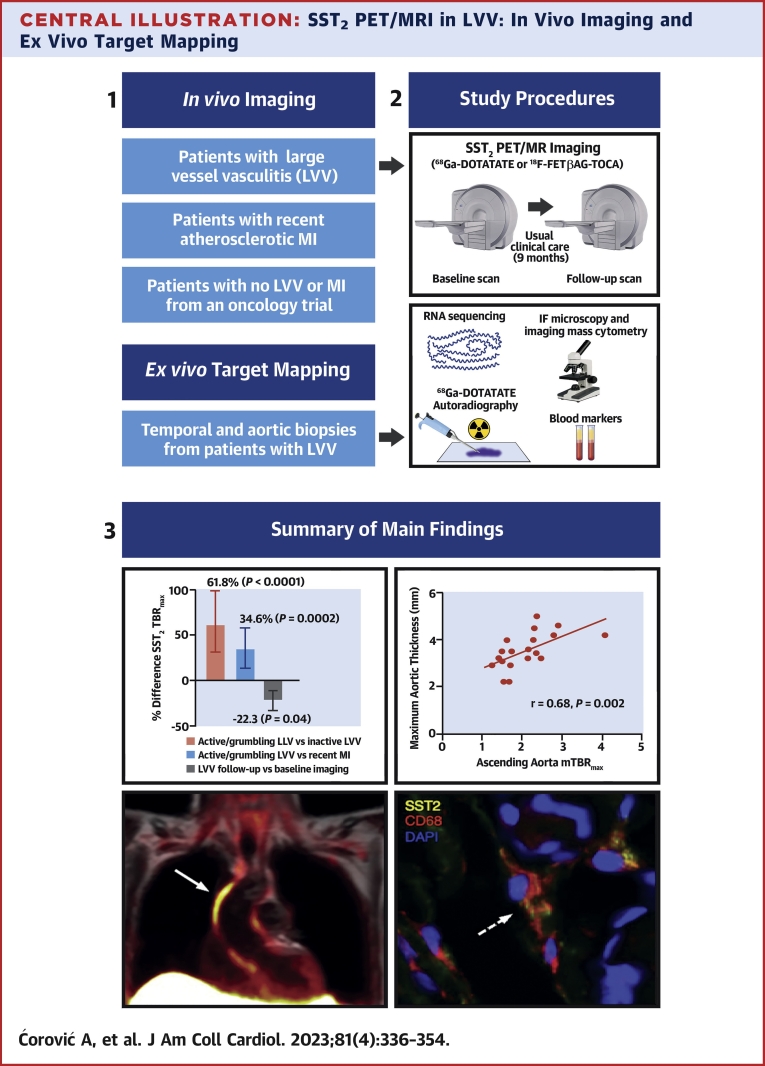
SST_2_ PET/MRI in LVV: In Vivo Imaging and Ex Vivo Target Mapping Patients with large vessel vasculitis (LVV) and recent atherosclerotic myocardial infarction (MI) underwent somatostatin receptor 2 (SST_2_) positron emission tomography/magnetic resonance imaging (PET/MRI) in a prospective observational cohort study. In parallel, ex vivo mapping of the imaging target was performed using RNA sequencing, histology, and autoradiography. The research methods and main study findings are summarized. Arterial SST_2_ signal **(arrow)** measured by the maximum tissue-to-background ratio (TBR_max_) using PET/MRI accurately differentiated patients with active/grumbling LVV from those with inactive LVV and recent MI, as well as control subjects. There was also a strong correlation between SST_2_ mean TBR_max_ and para-aortic thickening in LVV patients. SST_2_ expression was identified in macrophages **(dashed arrow)**, pericytes, and perivascular adipocytes with arterial specimens from patients with LVV. DOTATATE = DOTA-(Tyr3)-octreotate; FET = fluoroethyltriazole; IF = immunofluorescence.

### The unmet need for an inflammation-specific PET tracer for LVV

The European Alliance of Associations for Rheumatology future research agenda highlights the need to study PET ligands specifically targeted to immune cells.^1^ Although ^18^F-FDG PET is an important component of the diagnostic workup of patients with suspected LVV, it may be less useful for tracking response to therapy or monitoring long-term disease activity. Numerous studies have reported a discordance between the clinical response to treatment and ^18^F-FDG PET findings, with a high percentage of scans interpreted as active LVV despite patients achieving clinically defined disease remission.^6^, ^7^, ^8^, ^9^ Whether residual ^18^F-FDG activity in clinical remission patients is a marker of future relapse risk is unknown.^6^^,^^10^ However, a lack of association between ^18^F-FDG uptake and acute phase markers, arterial wall thickening, and late gadolinium enhancement on MRI has also been reported.^6^^,^^11^^,^^12^ It remains unclear whether ^18^F-FDG uptake during clinical remission reflects subclinical vasculitis, chronic vascular remodeling, concomitant atherosclerosis, or another factor.

### Could SST_2_ PET/MRI be useful for assessing disease activity in LVV?

We found that SST_2_ PET/MRI could accurately differentiate patients with clinically active/grumbling LVV from those with inactive LVV as well as aortic inflammation caused by recent atherosclerotic MI. SST_2_ PET signal was also correlated with periaortic thickening on MRI, another important marker of disease activity. Aside from our previous case report,^13^ the only other publication about SST_2_ imaging in vasculitis evaluated somatostatin receptor scintigraphy for detecting pulmonary and nasopharyngeal involvement in antineutrophilic cytoplasmic antibody–associated vasculitis.^14^ TBR values reported for atherosclerosis in our previous PET/CT study are not directly comparable to the present study because of differences in scanner type and image reconstruction.^2^

### Is there a role for therapeutic monitoring with SST_2_ PET?

Repeat scanning showed that SST_2_ PET/MRI was able to track the clinical response to LVV therapy (or lack thereof), indicating that it could provide a means of identifying refractory or residual arteritis after treatment. Importantly, there was good scan-scan repeatability of arterial TBR measurements. In contrast, the effect of treatment on CRP and other blood inflammatory markers was less consistent. Monitoring disease activity in patients treated with tocilizumab is another clinical need, because CRP is reduced directly by interleukin-6 inhibition and is, therefore, not useful for monitoring inflammation in the arterial wall. In a small, exploratory subgroup of patients treated with tocilizumab in this study, changes in arterial SST_2_ PET signals were again consistent with individual clinical responses to this therapy. A larger randomized clinical trial is needed to confirm if SST_2_ PET could be useful for on-treatment monitoring with tocilizumab or other agents.

### Target validation of SST_2_ in LVV

Both *SSTR2* gene expression and the presence of SST_2_ receptors were confirmed in macrophages within temporal artery biopsy samples from patients with GCA using multiple methods. Although we did not formally quantify costaining, the histologic findings were nonetheless consistent and reproducible across multiple samples. These findings are also consistent with a previous study that showed SST_2_ staining in CD68^+^ macrophages in sarcoid granulomas and a temporal artery biopsy specimen from 1 patient with GCA.^15^

We also found that in patients with LVV, SST_2_ PET signals could additionally originate from pericytes and adipocytes within inflamed periadventitial tissue. Somatostatin receptor expression has been identified in pericytes from patients with interstitial lung disease^16^ and retinal disease,^17^ and avid ^68^Ga-DOTATATE uptake has also been reported in a patient with a rare metastatic hemangiopericytoma.^18^ Although endothelial cell *SSTR2* expression has also been reported,^19^ data from large publicly available RNAseq databases show very low or no *SSTR2* expression in endothelial cells (eg, European Blueprint Study,^20^ Tabula sapiens human cell atlas^21^), which is consistent with our findings. *SSTR2* expression in adipose tissue is corroborated by data from the Human Protein Atlas.^22^

### Study limitations

As the first proof-of-concept study to evaluate SST_2_ in LVV, there are several limitations to acknowledge, including a nonrandomized observational study design, modest sample size, and lack of head-to-head comparison with ^18^F-FDG. Given the clinical importance of ^18^F-FDG PET imaging in LVV despite its known limitations, identifying an alternative imaging target with as much promise as SST_2_ represents a breakthrough. However, our study was not designed to directly evaluate SST_2_ PET against the current reference standard because it was first necessary to establish feasibility and to lay the translational groundwork. For grading of clinical LVV disease activity, the PGA score was used because there are no other validated disease activity measures for both GCA and TAK.^23^ Images from a first-in-human oncology trial were used as controls for arterial ^18^F-FET-βAG-TOCA uptake because this is the only other study that has used this tracer. Although semiquantitative TBR metrics were used instead of visual assessment scores because of high physiologic liver uptake, precluding its use as a background reference, TBR is an established method for vascular imaging research. Finally, the participant dropout rate before follow-up imaging was higher than expected because of the first wave of the COVID-19 pandemic.

## Conclusions

Somatostatin receptor PET/MRI using repurposed radioligands such as ^68^Ga-DOTATATE or ^18^F-FET-βAG-TOCA could offer a useful clinical adjunct for the diagnosis and monitoring of disease activity and therapeutic efficacy in LVV.

Perspectives **COMPETENCY IN PATIENT CARE AND PROCEDURAL SKILLS:** Although ^18^F-FDG PET can identify large vessel vasculitis, nonspecific uptake may occur during clinical remission. Somatostatin receptor 2 PET/MRI is an alternative method for assessment of inflammatory disease activity in LVV.

**TRANSLATIONAL OUTLOOK:** Further studies are needed to compare the sensitivity and specificity of these diagnostic modalities across the clinical spectrum of disease activity and responses to treatment.

## Funding Support and Author Disclosures

This work was funded by grants to Dr Tarkin from the Wellcome Trust (Clinical Research Career Development Fellowship 211100/Z/18/Z), the National Institute for Health Research (NIHR) Imperial Biomedical Research Centre (BRC); and the British Heart Foundation (BHF) (Clinical Research Training Fellowship for Dr Ćorović [FS/CRTF/20/24035]). This work was additionally supported by the Cambridge BHF Centre of Research Excellence (18/1/34212) and the Cancer Research UK Cambridge Centre (A25177). For the purpose of open access, the lead author has applied a CC BY public copyright license to any Author Accepted Manuscript. The views expressed are those of the author(s) and not necessarily those of the NHS, the NIHR, or the Department of Health and Social Care. Dr Nus; authors Imaz and Lambert; Dr Frontini (FS/18/53/33863); Dr Davenport (TG/18/4/33770); and Drs Huang, Mallat, Dweck, Newby, and Bennett are supported by the BHF. Author Zulcinski is supported by the European Union’s Horizon 2020 Research and Innovation Programme (Marie Skłodowska-Curie grant agreement no. 813545). Drs Jayne, Rassl, and Graves are supported by the NIHR Cambridge BRC. Dr Fayad is supported by the National Institutes of Health/National Heart, Lung, and Blood Institute (R01HL135878). Dr Reynolds is supported by the Wellcome Trust. Dr Morgan is supported by the Medical Research Council (MRC) (MR/N011775/1), the NIHR Leeds BRC, the NIHR Leeds Medtech, and In Vitro Diagnostics Co-operative as well as an NIHR Senior Investigator award. Dr Aboagye acknowledges support from Imperial Experimental Cancer Research Centre and MRC (MR/J007986/1, MR/N020782/1); and is an inventor on the patent that developed the ^18^F-FET-βAG-TOCA radiotracer. Dr Peters is supported by a UK Research and Innovation Fellowship at Health Data Research UK (MR/S004068/2). Dr Rudd is partly supported by the NIHR Cambridge BRC, the BHF, the Higher Education Funding Council for England, the Engineering and Physical Sciences Research Council, and the Wellcome Trust. Drs Gopalan, Maughan, Pericleous, Barwick, Aboagye, Peters, and Mason acknowledge support from the NIHR Imperial BRC. The authors have reported that they have no relationships relevant to the contents of this paper to disclose.
