# Supplementary material for: Somatostatin Receptor PET/MR Imaging of Inflammation in Patients With Large Vessel Vasculitis and Atherosclerosis
Source: J Am Coll Cardiol. 2023 Jan 31;81(4):336–54. doi: 10.1016/j.jacc.2022.10.034 (PMC9883634; doi:10.1016/j.jacc.2022.10.034)
Supplement: Supplemental Data [file mmc1.docx]

**SUPPLEMENTAL MATERIAL**

**Somatostatin Receptor PET/MR Imaging of Inflammation in Patients With Large Vessel Vasculitis and Atherosclerosis**

**Author contributions:**J.M.T. conceived, designed, and directed the study, collected and analyzed data, and wrote the manuscript with input from all authors. J.H.F.R., J.E.P., and J.C.M. conceived and designed the study. A.C. enrolled participants, analyzed images, and performed autoradiography. C.W., J.C.M., J.E.P., N.J., D.R.J., S.P.H., and P.A.C. identified and/or enrolled participants. D.G., L.A., T.B., R.M., T.D.F., M.J.G., F.G., E.O.A., S.D., M.R.D., D.E.N., and Z.A.F. contributed to the planning and interpretation of imaging data. Y.H. performed statistical analyses. E.O.A. developed the ^18^F-FET-bAG-TOCA tracer. E.O.A. and S.D. provided the oncological PET/CT dataset. A.F.D. and D.R. reported the histology and provided arterial specimens. M.R.B., A.P.D., Z.M., J.C.M., and G.H. designed experiments and provided lab facilities. M.N., A.U., and D.B. designed and performed histological experiments. M.P., R.T.M., and C.P. designed and performed ELISA assays. M.I., M.Z., G.R., H.J., J.L., M.I., M.F., and A.W.M. performed and/or analyzed RNAseq data. All authors discussed and interpreted the findings and contributed to the overall scientific content. E.O.A. provided access to the ^18^F-FET-βAG-TOCA tracer.

**Supplemental Methods**

1. Study inclusion/exclusion criteria, pg. 3
2. Grading of LVV clinical disease activity, pg. 4
3. Measurement of inflammatory blood markers, pg. 4
4. Imaging, pg. 4-5
5. Image analysis, pg. 5-6
6. RNAseq, pg. 6-8
7. Immunofluorescence microscopy, pg. 8
8. Autoradiography, pg. 8-9
9. Imaging mass cytometry, pg. 9-10
10. Sample size calculation, pg. 10
11. References, pg. 10

**Supplemental Figures**

Supplemental Figure 1. Indian Takayasu clinical activity score, pg. 11

Supplemental Figure 2. Comparison of SST_2_ PET imaging tracers, pg. 12

Supplemental Figure 3. SST_2_ PET in LVV vs aortic atherosclerosis, pg. 13

Supplemental Figure 4. ^18^F-FET-βAG-TOCA PET imaging in control subjects, pg. 14

Supplemental Figure 5. SST_2_ vs. ^18^F-FDG PET imaging, pg. 15

Supplemental Figure 6. Scan-scan repeatability, pg. 16

Supplemental Figure 7. Associations with clinical and biochemical markers, pg. 17

Supplemental Figure 8. SST_2_ PET vs aortic wall thickness, pg. 18

Supplemental Figure 9. Cell clusters for single-nuclei RNAseq, pg. 19

Supplemental Figure 10. RNAseq cluster analysis markers, pg. 20

Supplemental Figure 11. SST_2_ staining in aortitis, pg. 21

**Supplemental Tables**

Supplemental Table 1. Summary of TBR values, pg. 22

Supplemental Table 2. Summary of regression analysis data, pg. 23

Supplemental Table 3. Summary of data for participants who underwent repeat scanning, pg. 24

Supplemental Table 4. Univariable regression analysis of potential confounding factors, pg. 25

Supplemental Table 5. Correlation of *SSTR2* and *CD68* expression, pg. 26

Supplemental Table 6. Details of arterial specimens used for histology and other analyses, pg. 27

Supplemental Table 7. Number of nuclei per cell cluster in single-nuclei RNAseq, pg. 28

**SUPPLEMENTAL METHODS**

**PITA study**

*Inclusion Criteria:*

- Male or female participants >18 years old
- Able to give written, informed consent and to lie flat
- Either:

1. New suspected clinical diagnosis or acute flare of large vessel vasculitis (LVV; Giant-cell arteritis [GCA] or Takayasu arteritis [TAK]) within ~1 week of treatment initiation, or
2. LVV and a clinical indication for ^18^F-FDG PET-CT scan determined by the referring physician, or
3. Undergoing surgery for LVV, or
4. Prior evidence of LVV by imaging that was treated, with no current clinical signs or symptoms of active disease

*Exclusion Criteria:*

- Women of child-bearing potential not using adequate contraception
- Contraindication to MRI scanning
- Contrast allergy or contrast-nephropathy
- Chronic kidney disease (eGFR <30 mL/min/1.73 m^2^)
- Any medical condition, in the opinion of the investigator, that prevents the participant from lying flat during scanning or from participating in the study
- History of recent malignancy deemed relevant to the study by the investigator

**RIPPLE study**

*Inclusion Criteria:*

- Male or female participants >18 years old
- Able to give written, informed consent and to lie flat
- First presentation of myocardial infarction (MI) within ~2 weeks
- At least mild nonculprit coronary artery disease on angiography, managed medically

*Exclusion Criteria:*

- Women of childbearing potential not using adequate contraception
- Contrast allergy or contrast nephropathy
- Uncontrolled atrial fibrillation
- Chronic kidney disease (eGFR <30 mL/min/1.73 m^2^)
- Any medical condition that, in the opinion of the investigator, prevents the participant from lying flat during scanning, or from participating in the study
- Uncontrolled chronic inflammatory disorder
- History of recent malignancy deemed relevant to the study by the investigator
- Current use of systemic corticosteroids
- Previous coronary artery bypass grafting surgery (CABG) or percutaneous coronary intervention (PCI) before the index event
- Contraindication to coronary angiography
- Requires CABG or staged nonculprit artery PCI
- Previous history of myocardial infarction or heart failure

**Grading of LVV clinical disease activity.** Clinical LVV activity status was determined independent of the study findings according to the Physician Global Assessment (PGA) by 3 rheumatologists specializing in vasculitis, who were either directly involved in patient care or had full access to clinical notes. The initial assessment was made by the treating rheumatologist. For borderline cases, a consensus expert opinion was agreed. LVV disease activity was graded as “active”: newly diagnosed LVV or acute flare requiring treatment escalation; “grumbling”: low-grade residual arteritis; or “inactive”: disease in remission. Patients with TAK were also graded using the Indian Takayasu Clinical Activity Score (ITAS) and ITAS-CRP.^1^ Patients with TAK were considered “active” with ITAS of ≥2 and symptoms; “grumbling” with ITAS of 1, CRP of >0, and persistent symptoms; and “inactive” if ITAS = 0 and no symptoms or signs of disease flare.

**Measurement of inflammatory blood markers.** Venous blood was collected in EDTA, heparin, and SST-II tubes for measurement of systemic inflammatory markers. C-reactive protein (CRP) and erythrocyte sedimentation rate (ESR) were measured using validated clinical assays in the biochemical laboratories of Cambridge University Hospitals NHS Trust and Imperial College Healthcare NHS Trust. For measurement of interleukin 6 (IL-6) and Pentraxin-3 (PTX3), plasma and serum were separated by centrifugation and stored at -80°C. Enzyme-linked immunosorbent assays (ELISA) were performed using commercially available kits (R&D Systems DuoSet). The ranges of calibrator curves and sample dilutions were determined through initial optimization using the plasma patients and healthy controls (n=4).

**Imaging.** Patients with LVV underwent baseline and follow-up SST_2_ imaging using ^68^Ga-DOTATATE (in Cambridge) or ^18^F-FET-βAG-TOCA (at Imperial College) on an integrated PET/MR scanner (SIGNA, GE Healthcare). Radiopharmaceutical preparation of ^68^Ga-DOTATATE was performed using Netspot (Advanced Accelerator Applications) and ^18^F-FET-βAG-TOCA as previously described,^2^ with radiochemical purity confirmed. The target injected activity was 250 MBq, and circulation time was 50 min for both tracers (determined by prior dynamic imaging studies), after which two sequential 30-min acquisitions covering the thoracic aorta and head/neck vessels were performed. In patients with MI, a single bed position was used, covering the heart and ascending aorta. Static PET images were reconstructed from list mode data using iterative time-of-flight (256x256 matrix, Q.Clear b=350) and a free-breathing two-point DIXON MR imaging sequence for attenuation correction. Anatomical 3T MR imaging sequences included 3-plane aortic breath-held proton-density weighted, blood-suppressed single-shot fast-spin echo (5-mm slice thickness), 3D carotid TOF MR angiography (3-mm slice thickness), T1-weighted 3D fast spin echo with fat-suppressed (1.4-mm slice thickness), and 3D contrast-enhanced MR angiography using a gadolinium-based contrast agent (Gadovist).

Patients with an oncological indication for scanning underwent multibed whole body ^18^F-FET-βAG-TOCA imaging (maximum injected activity 165 MBq) as part of a separate trial, using a PET/CT scanner (Biograph, Siemens) with ordered-subsets expectation maximization reconstruction (3 iterations, 21 subsets).^2^

**Image analysis.** Images were analyzed using open-source medical imaging software (Horos, v3.3.6), with the readers blinded to clinical details. 2D regions of interest (ROIs) were drawn around the outer vessel boundaries on consecutive coregistered PET/MR imaging slices (thickness: 5 mm aortic; 3 mm carotids) orientated in the transaxial plane for the thoracic aorta, proximal aortic arch vessels, and carotid and vertebral arteries. Arterial radioactivity concentration measured as standardized uptake value was normalized by mean blood pool activity in the superior vena cava or internal jugular vein to derive mean (m) and most diseased segment (mds) maximum tissue-to-blood ratios (TBR) as per established recommendations for vascular imaging,^3^ in the index vessel, thoracic aorta, and all vessels combined. The mds was defined as the highest arterial TBR_max_ slice, averaged with contiguous slices above and below. The index vessel was the artery with the highest mTBR_max_. The intra- and interobserver repeatability of these methods has previously been demonstrated using ^68^Ga-DOTATATE.^4^

As control subjects were imaged using PET/CT rather than PET/MR and different reconstruction methods applied, vascular uptake was assessed visually by 2 experienced radiologists using background activity in the mediastinum for reference.

Maximum aortic wall thickness (mm) was measured by MRI in the transaxial plane using blood-suppressed images.

**Bulk RNAseq.** RNA was extracted from formalin-fixed paraffin-embedded (FFPE) temporal artery specimens from participants of the UK GCA Consortium using the Ambion RecoverAll Total Nucleic Acid Extraction for FFPE kit. The protocol was modified such that 30 × 5-µm sections were cut from each block; deparaffinized by immersing slides in xylene (4 × 5 min); washed with ethanol (4 × 5 min) to remove the xylene; and subsequently rehydrated using 75% ethanol, 50% ethanol, and finally water. The protocol from the kit was then followed using 200 µL digestion buffer, overnight incubation at 50°C, and RNA eluted in 60 µL of nuclease-free water. RNA was then quantified using Qubit, and DV200 (a measure of what proportion of the RNA is above 200 bp) was measured on Tapestation. Samples with a DV200 of less than 30% were considered degraded and not used for library preparation. 100 ng of all samples with a DV200 value above 30% was used to generate the library using Illumina’s RNA Exome Library Preparation kit. The final library was quantified using QuantIT and checked on Tapestation for the correct insert size and presence of any adapter contamination.

RNAseq workflow included quality control (FastQC), trimming (Trimmomatic), and reads mapping and quantification (Salmon) with index built on ensembl.org/pub/release-94/fasta/homo_sapiens/cdna/Homo_sapiens.GRCh38.cdna.all.fa.gz. Postprocessing of transcript- and gene-level counts was performed using “tximport” Bioconductor package.

**Single-cell RNAseq.** Donors were recruited from the Freeman Hospital in Newcastle-Upon-Tyne, UK. Temporal artery biopsy samples were processed immediately after surgery. Temporal artery tissue was mechanically digested with a scalpel into 2- to 3-mm fragments before being placed in PBS with Collagenase type I (Sigma, C0130, 400U/ml), Collagenase type XI (Sigma, C7657, 120U/ml), hyaluronidase (clinical grade, 60U/ml), DNase (Sigma, 11284932001, 60U/ml) in a water bath at 37 degrees for one hour. The sample was then filtered and treated with an RBC lysis buffer (eBioscience). Cells were then washed again and counted. The sample was loaded on to the 10x Chromium controller using the Chromium NextGEM Single Cell V(D)J Reagent kit v1.1. Gene expression libraries were prepared according to the manufacturer’s protocol (10X Genomics). Libraries were sequenced on an Illumina NovaSeq 6000 to achieve a minimum of 50,000 paired-end reads for gene expression.

**Single-nuclei RNAseq.** Temporal and carotid artery tissue samples were snap-frozen in liquid nitrogen before being embedded in Optimal Cutting Temperature compound (OCT). For nuclei extraction, 7 × 40-µM-thick sections were cut and nuclei purified using established methods.^5^ Briefly, 1 mL nuclear extraction buffer (NEB; 20 mM Tris. pH8, 320 mM sucrose, 5 mM CaCl_2_, 3 mM MgAc_2_, 0.1 M Ethylenediaminetetraacetic acid [EDTA], 0.1% Triton-X-100, dH_2_O) supplemented with 0.1% RNase Inhibitor (Enzymatics, Y9240L) and 5 mg/mL DAPI (Sigma, D9564), was added to the OCT-embedded sections, the sample dissociated by pipetting (10-15× using a P1000 with a wide-bore tip) and disrupted using a Dounce homogenizer (Sigma-Aldrich, D8938) with 5 × pestle A and 20 × pestle B strokes. The solution was incubated on ice for 15 minutes to lyse cells before being passed through a 30-μM CellTrics filter, diluted in wash buffer (PBSE; 1× PBS, 1 mM egtazic acid [EGTA]) and centrifuged at 900*g* for 10 minutes at 4°C to pellet the nuclei. The pelleted nuclei were resuspended in PBSE supplemented with 1% BSA and counted before immediate submission for 10X processing. Nuclei (14-16,000 per reaction) were loaded onto the 10X Chromium X and processed according to the manufacturer’s protocols. The indexed cDNA libraries were sequenced on the Illumina NovaSeq6000.

**Immunofluorescence microscopy.** FFPE sections (8 µm) of temporal artery biopsies were first assessed using hematoxylin and eosin stain with light microscopy (Olympus BX51). SST_2_ staining was then assessed using immunofluorescence microscopy (Leica BD6000) in adjacent sections costained with primary antibodies for SST_2_ (Novus, NB300-157; 1:100) and CD68 (Dako, Clone PG-M1; 1:100), along with a nuclear DAPI counterstain. SST_2_ signal was amplified with biotinylated anti-rabbit IgG followed by streptavidin (BioLegend, AF488; 1:500). Methods for antigen retrieval, washing (PBS-Triton 0.3%) and blocking (3% BSA/20 mM MgCl/0.3% Tween/5% goat serum in PBS) with overnight incubation at 4°C were as previously described.^6^

**Autoradiography.** ^68^Ga-DOTATATE autoradiography was performed as previously described.^4^ Arterial biopsies were snap-frozen in liquid nitrogen and stored at -80°C. Consecutive 20 µm cryostat sections were cold mounted onto slides. After preincubation with HEPES (4-[2-hydroxyethyl]-1-piperazineethanesulfonic acid) 10 mM buffer, ^68^Ga-DOTATATE 10 nM solution was applied for 30 minutes at room temperature. Adjacent sections were incubated with a competing concentration of unlabeled DOTATATE (1 µM) to define regions of nonspecific binding. At the end of the experiment, sections were washed in Tris-HCL (pH 7.8), dipped in distilled H_2_O, and apposed to a phosphor screen. Radioactivity within sections was visualized using a Cyclone Storage Phosphor System (PerkinElmer) and OptiQuant image analysis software.

**Imaging mass cytometry.** Imaging mass cytometry was performed in FFPE temporal artery sections (8 µm) with FITC-conjugated SST_2_ (Novus, NB300-157), DAPI nuclear counterstain, and a panel of 10 additional metal-conjugated antibodies (CD3: Fludigm, 3170019D, 0.5 mg/mL; CD4, Fludigm, 3141017D, 25 µg/mL; αSMA: Fludigm, 3141017D, 0.5 mg/mL; CD31: Fludigm, 3151025D, 25 µg/mL; CD19: Fludigm, 3142014D, 25 µg/mL; CD45: Fludigm, 3152018D, 25 µg/mL; CD68: Fludigm, 3159035D, 25 µg/mL); CD80: Abcam, ab254579, 0.5 mg/mL; CD206: Abcam, ab64693, 0.5 mg/mL; NG2: Sigma-Aldrich, AB5320, 0.5 mg/mL).

FFPE sections were heated, dewaxed using m-xylene followed by descending grades of ethanol and rinsed in water prior to antigen retrieval for 20 minutes at 96°C using Tris-EDTA HIER buffer at pH 9.2, rinsed again with PBS, and then blocked with cell staining buffer for 45 minutes at room temperature. FITC-conjugated SST_2_ and metal conjugated antibodies were mixed at specific concentrations in cell-staining buffer with 0.5% BSA. The following day, slides were washed in 1% Tween and DPBS and incubated with 1:100 anti-FITC at 4°C overnight. MaxPar Ir-intercalator (500 mM at 1:2000) and DAPI (1:1000) were then added and slides left for 30 minutes at room temperature, and then rinsed in water.

Sections were scanned first through a Leica DMI4000 fluorescence microscope equipped with DAPI/FITC filter cubes and a Lumencor Spectra X light engine. The slide was then washed twice in ultrapure water and air-dried for 12 h before IMC imaging. IMC imaging was performed using a Fluidigm Hyperion imager instrument with ablation power set at 3. The images produced by IMC scanning were converted to ZARR format, and subsequently visualized and converted to .tiff format, using a custom pipeline.^7^ The IMC data-cube was realigned to the fluorescence images using the Fiji open-source software and the bigWarp plugin.^8^

**Sample size calculation.** Sample size was determined by an a priori power calculation based on pilot data from patients with recent MI who underwent ^68^Ga-DOTATATE PET/CT in a previous study, demonstrating aortic mTBR_max_ 2.42 (SD 0.36). Assuming a 20% difference in means (alpha=0.05, 2-sided) in aortic mTBR_max_ between active/grumbling LVV and recent MI, a sample size of n=27 per group was estimated to have 95% power, and n=17 per group would have 80% power.

**References:**

1. Misra R, Danda D, Rajappa SM, Ghosh A, Gupta R, Mahendranath KM, Jeyaseelan L, Lawrence A, Bacon PA. Development and initial validation of the Indian Takayasu Clinical Activity Score (ITAS2010). *Rheumatology* 2013;52:1795–1801.

2. Dubash SR, Keat N, Mapelli P, et al. Clinical Translation of a Click-Labeled 18F-Octreotate Radioligand for Imaging Neuroendocrine Tumors. *J Nucl Med* 2016;57:1207–1213.

3. Bucerius J, Hyafil F, Verberne HJ, et al. Position paper of the Cardiovascular Committee of the European Association of Nuclear Medicine (EANM) on PET imaging of atherosclerosis. *Eur J Nucl Med Mol Imaging* 2016;43(4):780-92.

4. Tarkin JM, Joshi FR, Evans NR, et al. Detection of Atherosclerotic Inflammation by 68Ga-DOTATATE PET Compared to [18F]FDG PET Imaging. *J Am Coll Cardiol* 2017;69:1774–1791.

5. Lake BB, Chen S, Hoshi M, et al. A single-nucleus RNA-sequencing pipeline to decipher the molecular anatomy and pathophysiology of human kidneys. *Nat Commun* 2019;10(1):2832.

6. Nus M, Martínez-Poveda B, Cardiovascular DM, 2016. Endothelial Jag1-RBPJ signalling promotes inflammatory leucocyte recruitment and atherosclerosis. *Cardiovasc Res* 2016;112(2):568-580.

7. González-Solares EA, Dariush A, González-Fernández C, Yoldaş AK, Sa’d Al M, Millar N, et al. The Imaging and Molecular Annotation of Xenografts and Tumours (IMAXT) High Throughput Data and Analysis Infrastructure. bioRxiv 2021; [online preprint] doi: 10.1101/2021.06.22.448403

8. Schindelin J, Arganda-Carreras I, Frise E. *et al.* Fiji: an open-source platform for biological-image analysis. *Nat Methods* 2012; 9(7), 676-82.


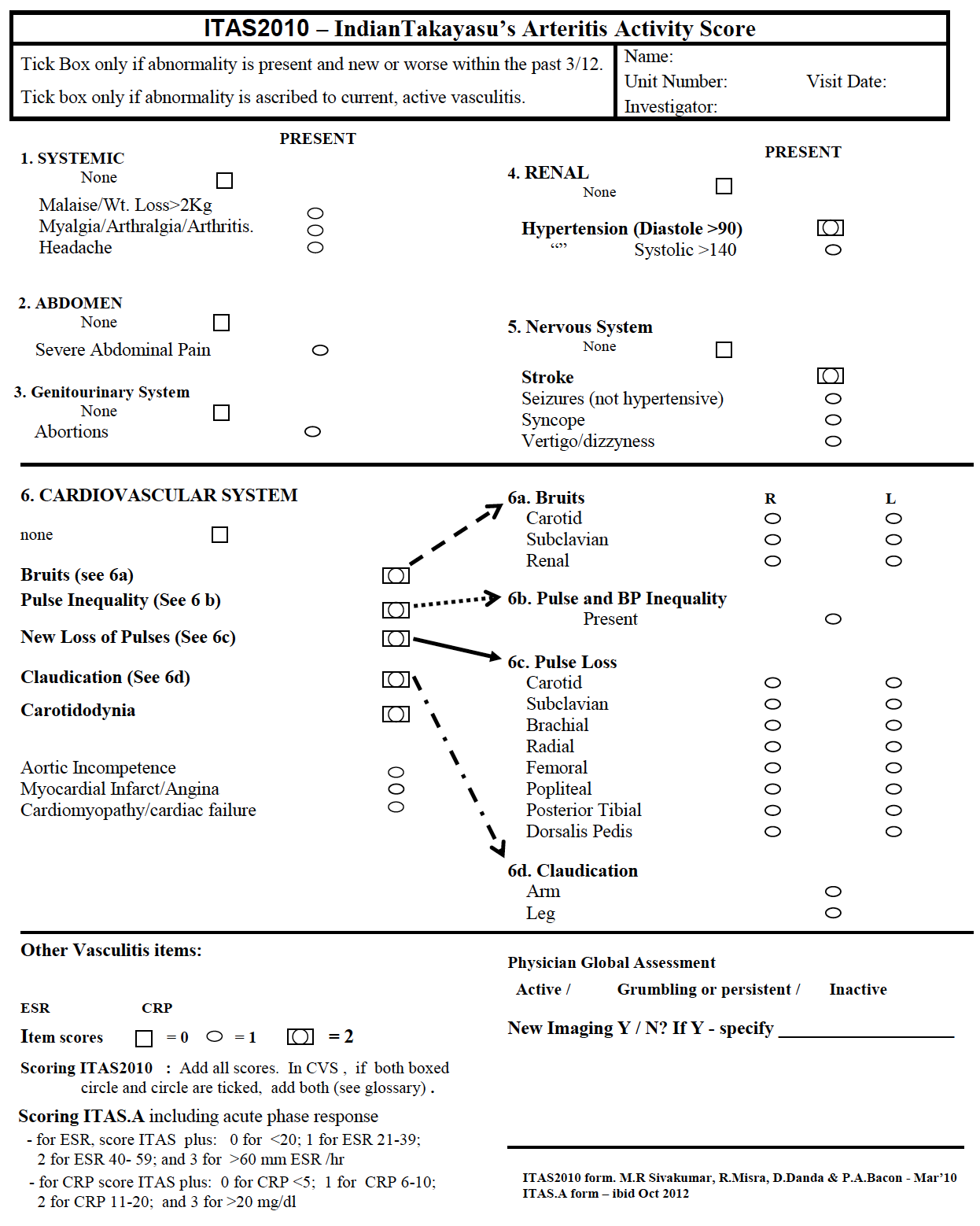
**SUPPLEMENTAL FIGURES**

**Supplemental Figure 1 | Indian Takayasu clinical activity score.** Summary of criteria used to calculate ITAS and ITAS-CRP.

**
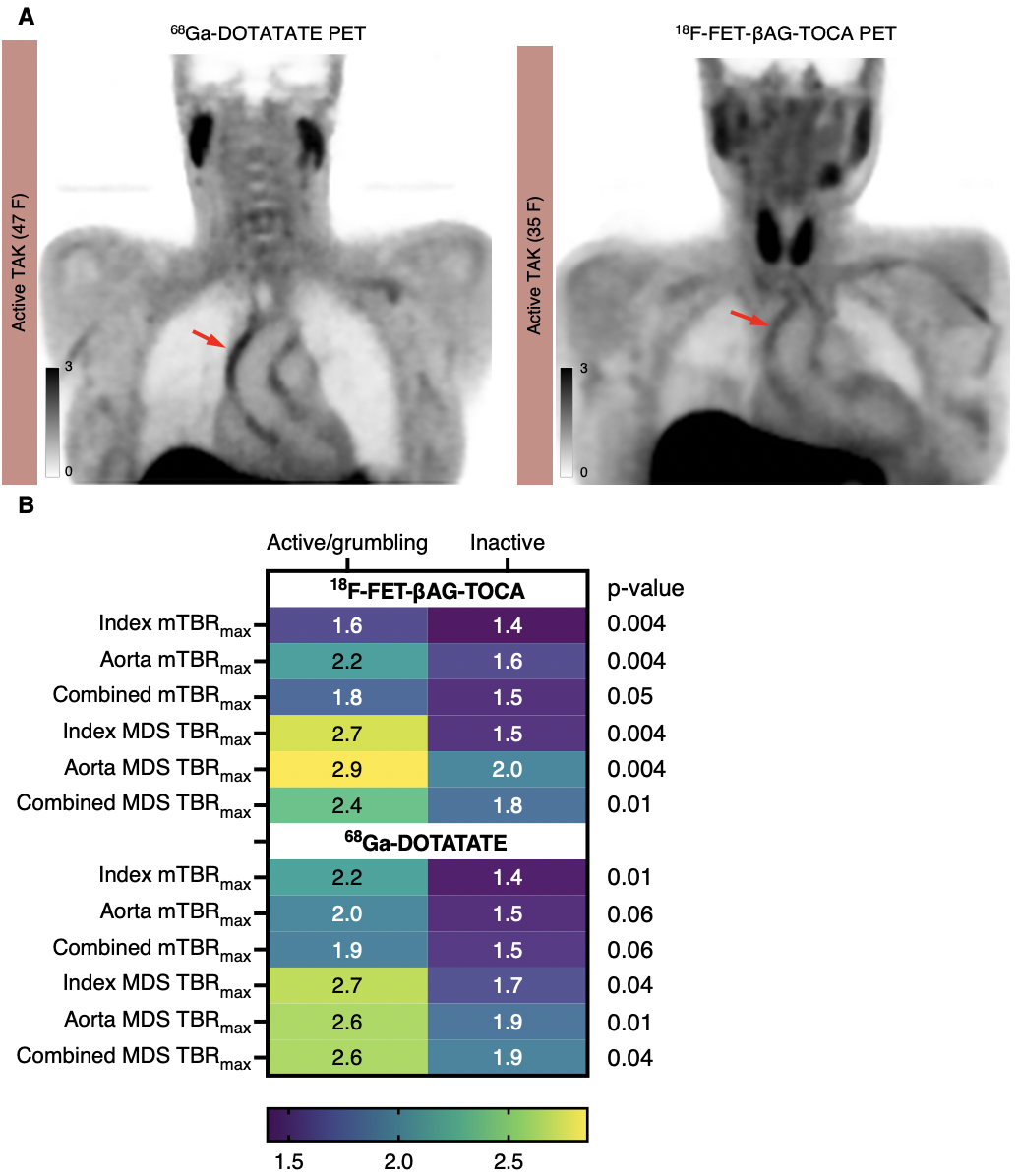
**

**Supplemental Figure 2 | Comparison of SST_2_ PET imaging tracers.** (**A**) Additional images of patients with active LVV demonstrating a similar ability of the two SST_2_ PET tracers (^18^F-FET-βAG-TOCA or ^68^Ga-DOTATATE) to identify aortic inflammation (red arrows); (**B**) Heatmap showing median mTBR_max_ and mdsTBR_max_ values for patients with active/grumbling LVV vs. inactive LVV grouped by SST_2_ tracer (^18^F-FET-βAG-TOCA or ^68^Ga-DOTATATE).

***
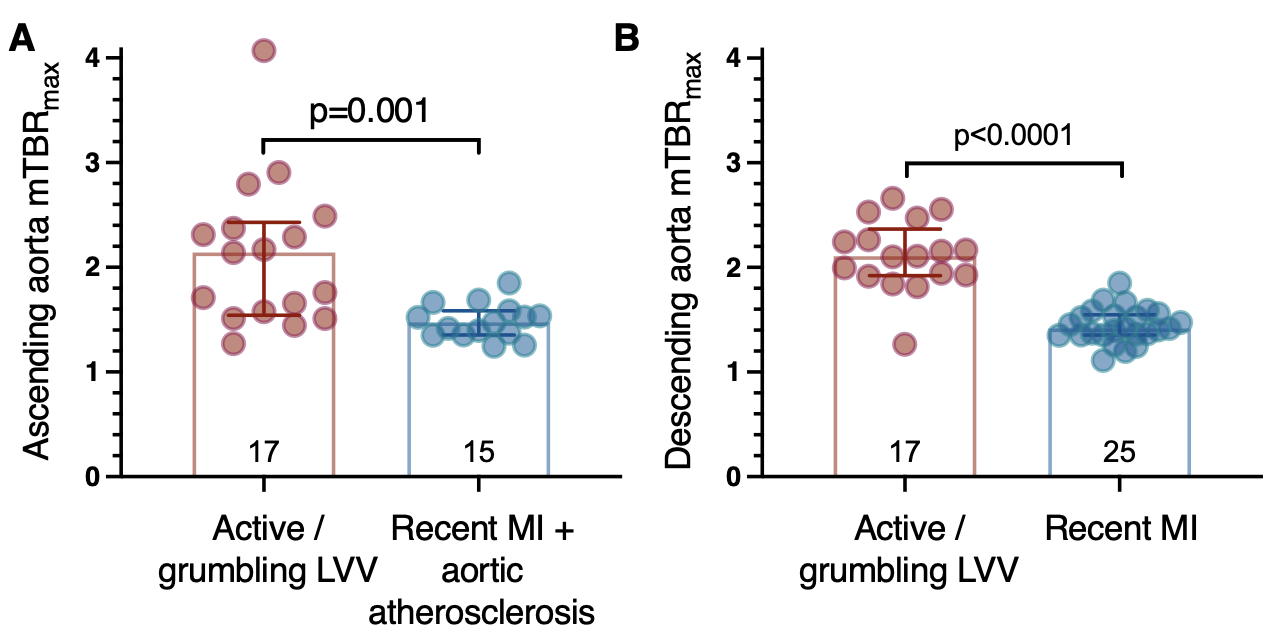
***

**Supplemental Figure 3 | SST_2_ PET in LVV vs aortic atherosclerosis.** Graphs comparing (**A**) ascending aortic SST_2_ PET mTBR_max_ in patients with active/grumbling LVV to those with recent MI and aortic atherosclerosis confirmed by CT angiography and (**B**) descending aorta SST_2_ PET mTBR_max_ in patients with active/grumbling LVV to those with recent MI. Error bars=median (IQR).

**Supplemental Figure 4 |** **^18^F-FET-βAG-TOCA PET imaging in control subjects.** PET images from all 9 control subjects without LVV or recent MI imaged as part of a prior oncology study of ^18^F-FET-βAG-TOCA imaging showing no aortic signal above background in the mediastinum**
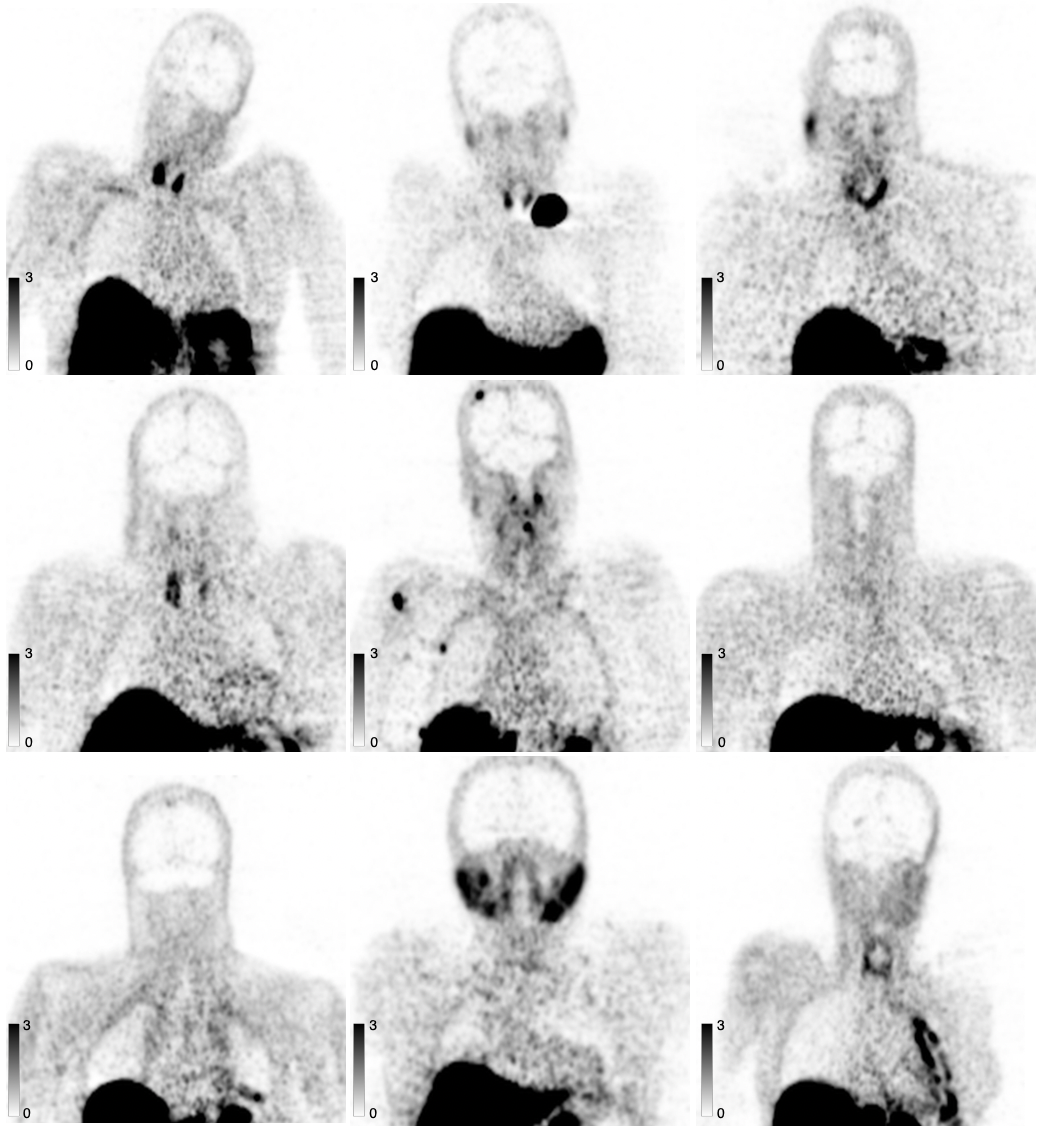
**.

***
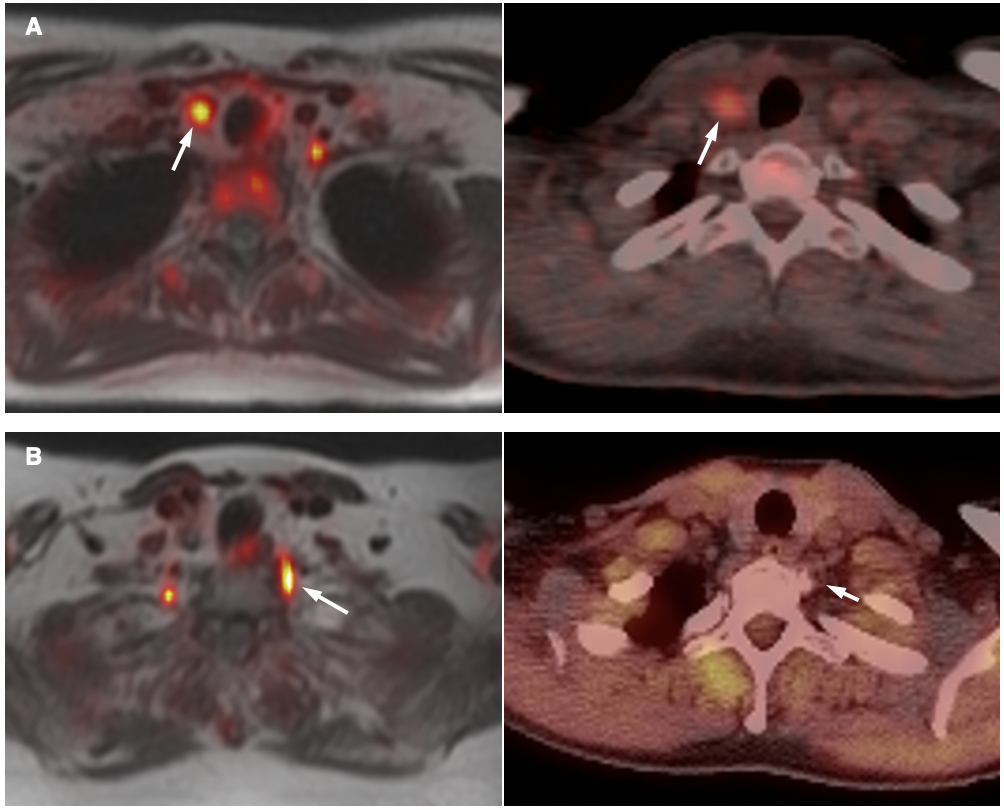
*Supplemental Figure 5 | SST_2_ vs. ^18^F-FDG PET imaging.** (**A**) ^18^F-FET-βAG-TOCA PET/MR (left) and ^18^F-FDG PET/CT (right) images (scan-scan interval 15 months) from a 27-year-old woman with grumbling TAK, both showing tracer uptake in right common carotid artery (arrows). In another patient (**B**), a 53-year-old woman with grumbling TAK, there is increased ^68^Ga-DOTATATE PET/MR (left) uptake in the vertebral arteries (arrows), which was not evident using ^18^F-FDG PET/CT (right; scan-scan interval 5 months).

**
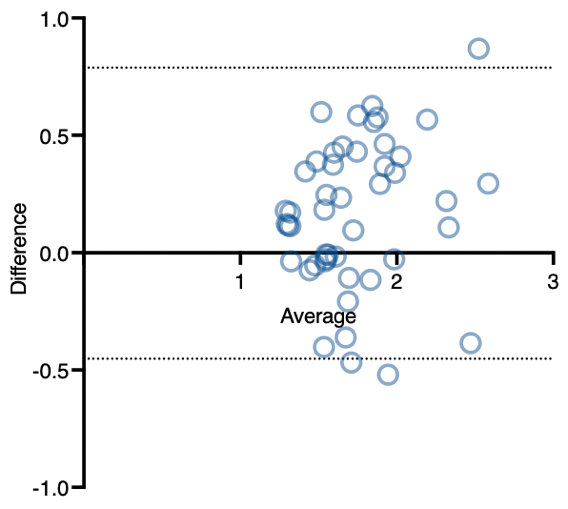
Supplemental Figure 6 | Scan-scan repeatability.** Bland-Altman plot comparing individual TBR_max_ values between baseline and follow-up scans in patients (n=4) with inactive disease and no change in treatment.

**
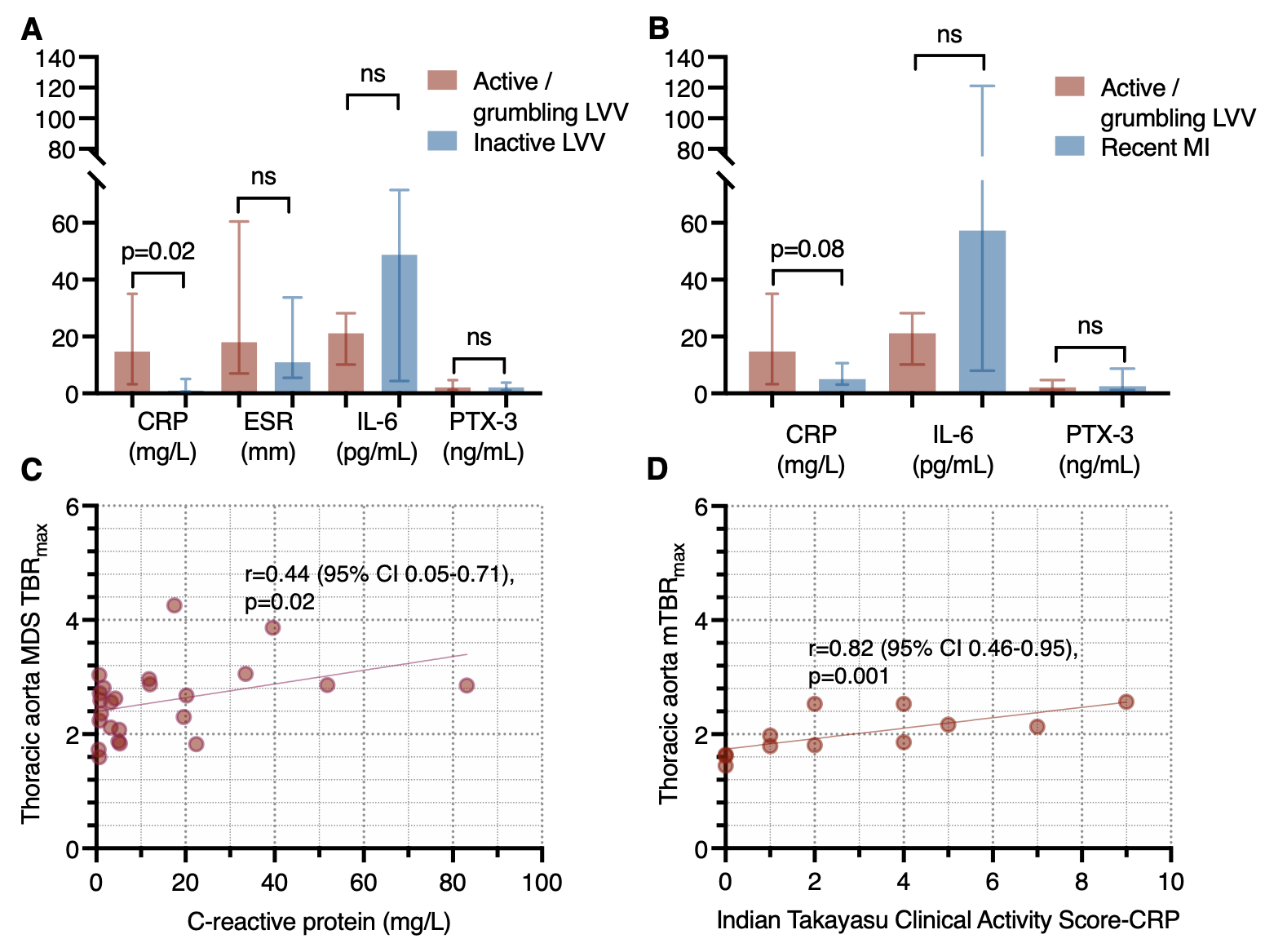
**

**Supplemental Figure 7 | Associations with clinical and biochemical markers.** Graphs showing levels of inflammatory blood markers in patients with active/grumbling LVV vs. (**A**) inactive LVV and (**B**) recent MI. CRP and IL-6 values excluded from panels **A** and **B** for 3 patients with active LVV and 1 patient with inactive LVV whose baseline treatment included tocilizumab. Scatter plots showing correlations of aortic SST_2_ PET/MR signals with (**C**) CRP and (**D**) ITAS-CRP score. Panel **D** includes only patients with Takayasu arteritis. Error bars=median (IQR).

**
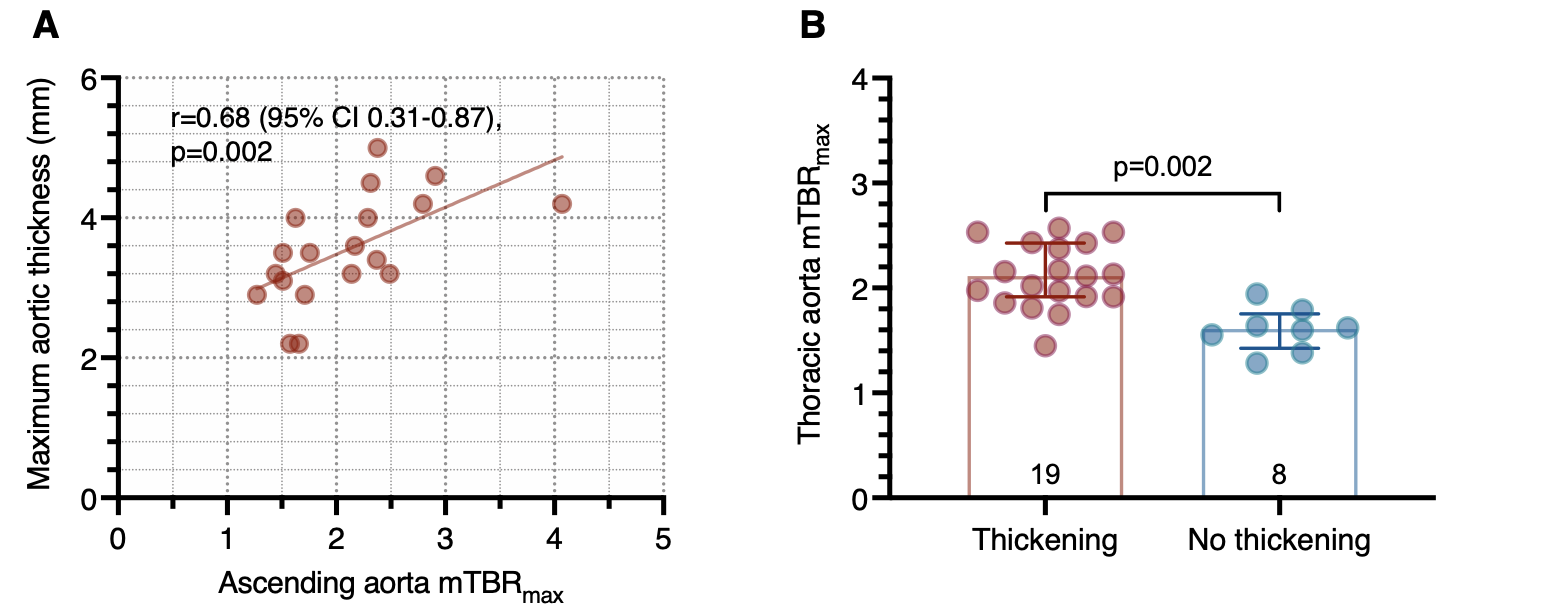
**

**Supplemental Figure 8 | SST2 PET vs aortic wall thickness.** (**A**) Scatter plot showing correlation of aortic SST_2_ PET/MR signals with wall thickness in LVV patients with aortic thickening (>2.2 mm);(**B**) Comparison of aortic SST_2_ PET mTBR_max_ in patients with and without aortic thickening on MR imaging. Error bars=median (IQR).

**
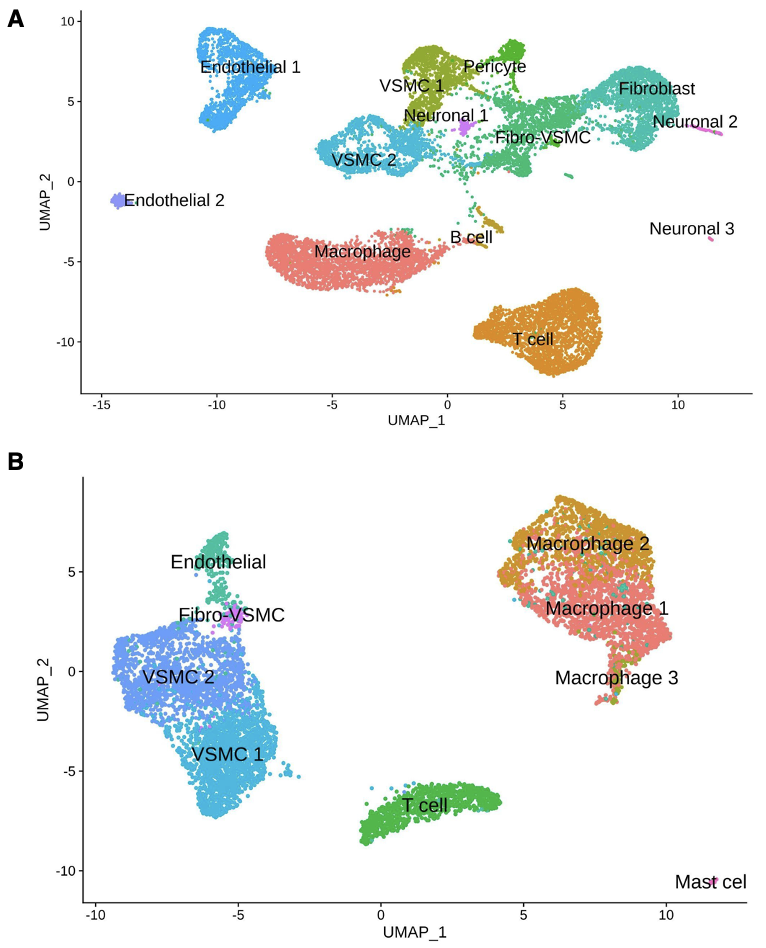
**

**Supplemental Figure 9 | Cell clusters for single-nuclei RNAseq.** U-MAPs showing cell clusters for (**A**) temporal artery and (**B**) carotid artery samples

***
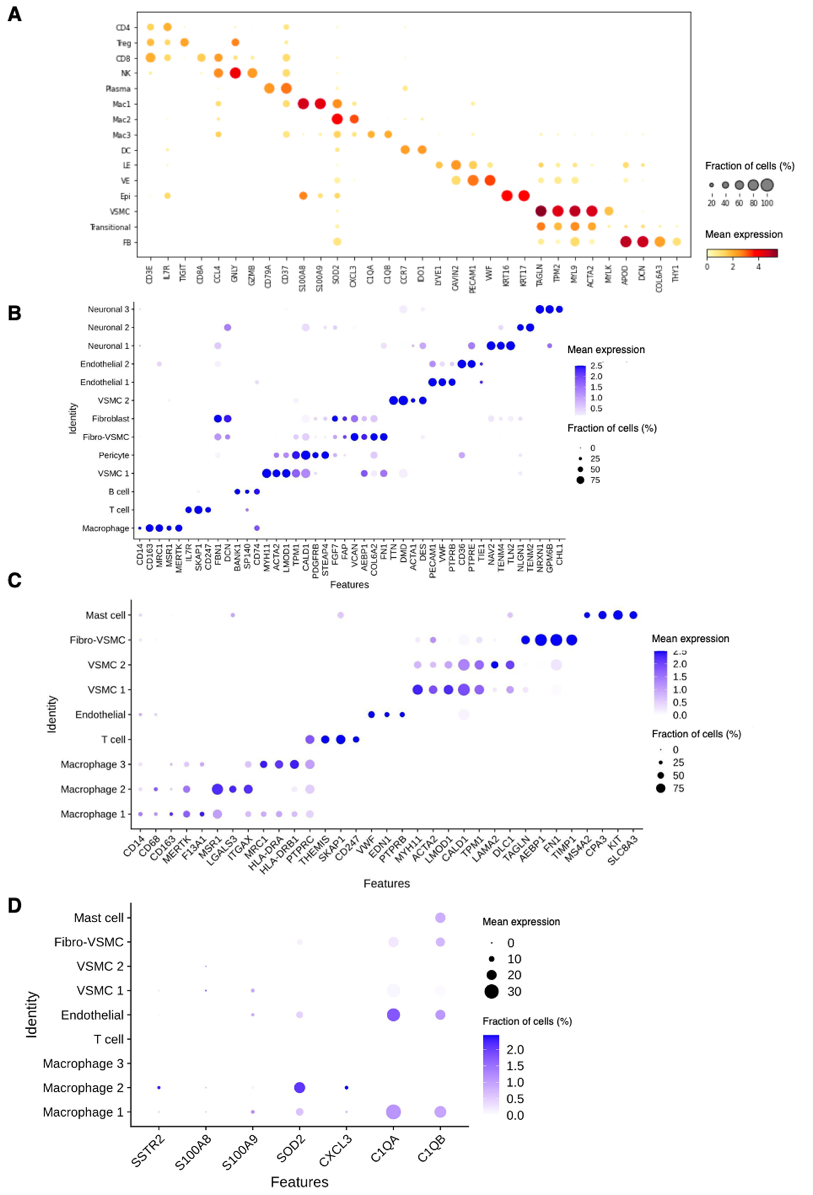
***

**Supplemental Figure 10 | RNAseq cluster analysis markers.** Plots showing cell markers used for (**A**) temporal artery single-cell RNAseq, (**B**) temporal artery single-nuclei RNAseq, and (**C, D**) carotid artery single-nuclei RNAseq cluster analyses.

**
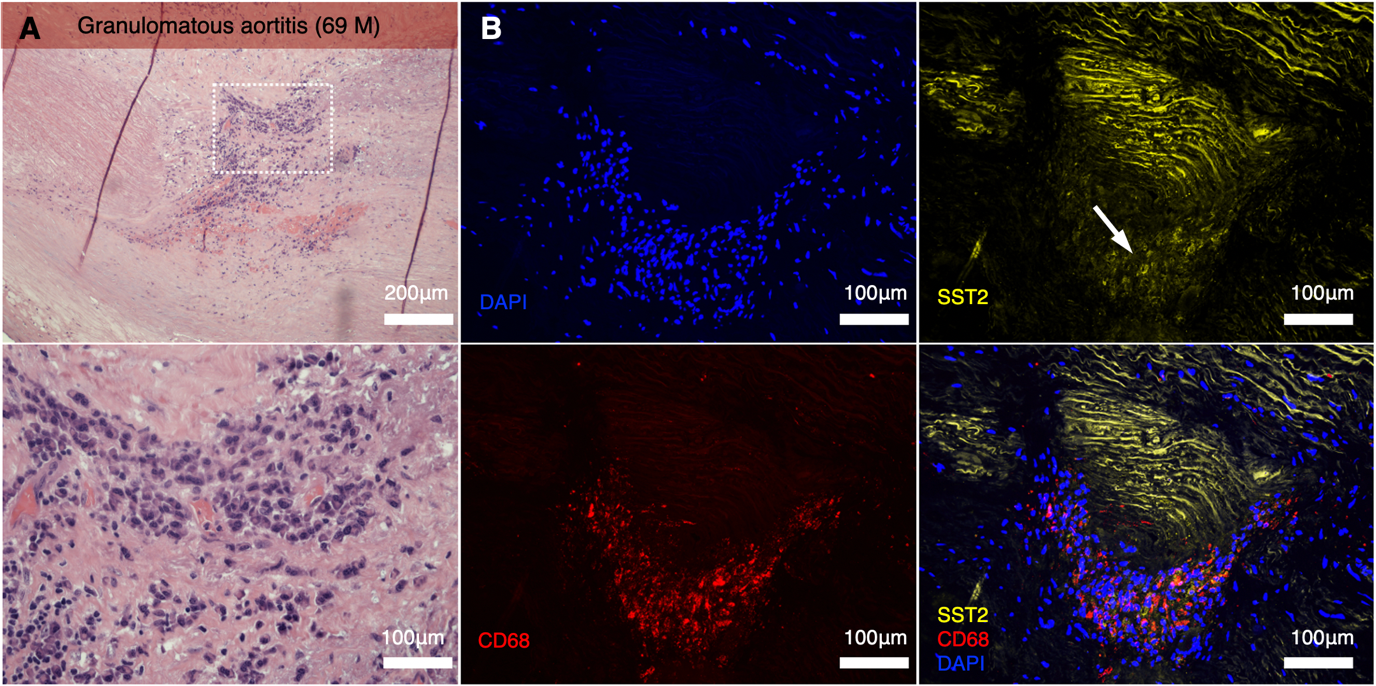
**

**Supplemental Figure 11 | SST_2_ staining in aortitis.** (**A**) Hematoxylin and eosin and (**B**) immunofluorescence images from a patient with granulomatous aortitis due to vasculitis who underwent aortic valve and ascending aortic replacement surgery, showing a cluster of punctate (arrow) costaining of SST_2_ and CD68^+^ macrophages within an inflamed region of the aorta, as well as linear nonspecific SST_2_ binding most likely to collagen or elastin.

**SUPPLEMENTAL TABLES**

**Supplemental Table 1 | Summary of TBR values**

**Supplemental Table 2 | Summary of regression analyses data**

**Supplemental Table 3 | Summary of data for participants who underwent repeat scanning**

**Supplemental Table 4 | Univariable regression analysis of potential confounding factors that could be associated with TBR_max_ values**

**Supplemental Table 5 | Correlation of *SSTR2* and *CD68* expression in bulk RNAseq dataset of temporal biopsies from patients with GCA**

*Pearson’s correlation of counts normalized by TMM (trimmed mean of M values) for all chromosomes

**Supplemental Table 6 | Details of arterial specimens used for histology and other analyses**

**Supplemental Table 7 | Number of nuclei per cell cluster in single-nuclei RNAseq**
